# Proteomics Perspectives in Rotator Cuff Research: A Systematic Review of Gene Expression and Protein Composition in Human Tendinopathy

**DOI:** 10.1371/journal.pone.0119974

**Published:** 2015-04-16

**Authors:** Maria Hee Jung Sejersen, Poul Frost, Torben Bæk Hansen, Søren Rasmussen Deutch, Susanne Wulff Svendsen

**Affiliations:** 1 Danish Ramazzini Centre, Department of Occupational Medicine, Regional Hospital West Jutland—University Research Clinic, Herning, Denmark; 2 Danish Ramazzini Centre, Department of Occupational Medicine, Aarhus University Hospital, Aarhus, Denmark; 3 Research Unit for Orthopaedics, Holstebro Regional Hospital, Holstebro, Denmark; 4 Institute of Clinical Medicine, Faculty of Health Sciences, Aarhus University, Aarhus, Denmark; 5 Department of Orthopaedic Surgery, Randers Regional Hospital, Randers, Denmark; Queen Mary University of London, UNITED KINGDOM

## Abstract

**Background:**

Rotator cuff tendinopathy including tears is a cause of significant morbidity. The molecular pathogenesis of the disorder is largely unknown. This review aimed to present an overview of the literature on gene expression and protein composition in human rotator cuff tendinopathy and other tendinopathies, and to evaluate perspectives of proteomics – the comprehensive study of protein composition - in tendon research.

**Materials and Methods:**

We conducted a systematic search of the literature published between 1 January 1990 and 18 December 2012 in PubMed, Embase, and Web of Science. We included studies on objectively quantified differential gene expression and/or protein composition in human rotator cuff tendinopathy and other tendinopathies as compared to control tissue.

**Results:**

We identified 2199 studies, of which 54 were included; 25 studies focussed on rotator cuff or biceps tendinopathy. Most of the included studies quantified prespecified mRNA molecules and proteins using polymerase chain reactions and immunoassays, respectively. There was a tendency towards an increase of collagen I (11 of 15 studies) and III (13 of 14), metalloproteinase (MMP)-1 (6 of 12), -9 (7 of 7), -13 (4 of 7), tissue inhibitor of metalloproteinase (TIMP)-1 (4 of 7), and vascular endothelial growth factor (4 of 7), and a decrease in MMP-3 (10 of 12). Fourteen proteomics studies of tendon tissues/cells failed inclusion, mostly because they were conducted in animals or in vitro.

**Conclusions:**

Based on methods, which only allowed simultaneous quantification of a limited number of prespecified mRNA molecules or proteins, several proteins appeared to be differentially expressed/represented in rotator cuff tendinopathy and other tendinopathies. No proteomics studies fulfilled our inclusion criteria, although proteomics technologies may be a way to identify protein profiles (including non-prespecified proteins) that characterise specific tendon disorders or stages of tendinopathy. Thus, our results suggested an untapped potential for proteomics in tendon research.

## Introduction

Tendinopathy may be understood as a clinical diagnosis designating tendon pain, which is often associated with tendon swelling and intratendinous changes[1]. Often, tendinopathy is used synonymously with tendinitis and tendinosis, but the last-mentioned terms may also be reserved for tendons with histopathologic findings[1]. Tendon specimens from humans and animals with tendinopathy often show histopathologic changes, which may precondition the tendon to rupture[2–5]. Clinical manifestations suspected to reflect rotator cuff tendon alterations (e.g. subacromial impingement syndrome) occur with a prevalence of 2–8% in general population samples[6–8], and increasing rates of subacromial decompression surgery have been reported[9–11]. Most likely, tendinopathy results from an interplay between intrinsic and extrinsic factors[12]. Intrinsic factors such as genetic polymorphisms[13–16], hypoxia[17], and apoptosis[18, 19] have been implicated together with extrinsic factors such as micro trauma[20, 21] and occupational biomechanical exposures[22, 23].

Tendon tissue research may be conducted at histological or molecular level. Histology refers to the microscopic study of tissues, whereas molecular studies focus on genes, transcripts, and proteins. Studies of genes give insights into factors, which may predispose individuals to tendon disorders, but the information is static. Studies of transcripts, on the other hand, provide information about current gene expression. Transcripts refer to messenger RNA (mRNA) produced by transcription of DNA and are usually detected using specific DNA probes or RNA primers. However, transcripts are poor indicators of protein levels because the resulting proteins may be modified or degraded, or accumulate in the extracellular matrix (ECM). Proteins in tendons are usually detected and quantified by means of specific antibodies. Due to their reliance on specific probes, primers, and antibodies, analyses of transcripts and traditional analyses of proteins do not allow identification of unexpected molecules.

The completion of the Human Genome Project in 2003 (www.ornl.gov/hgmis) enabled the emergence of OMICS technologies, which deal with the global characterisation of biological systems[24, 25]. OMICS classically includes genomics, transcriptomics, proteomics, and metabolomics, in order of increasing complexity of investigation (S1 Fig)[26]. Proteomics is the comprehensive study of protein composition, while metabolomics aims to explore metabolic activity via quantification of metabolites (including proteins). Proteomics and metabolomics primarily employ mass spectrometry (MS) in combination with bioinformatics.

MS-based proteomics identifies and quantifies proteins without a need for antibodies[25]; in a single specimen, hundreds of proteins may be identified and quantified. This technology has proved useful to identify candidate disease biomarkers and to generate novel hypotheses of disease mechanisms, most notably in cancer research[27]. In recent years, proteomics technologies have also found use in studies of musculoskeletal disorders, e.g., studies of osteoarthritic cartilage[28–31].

Based on a systematic review of the literature on gene expression and protein composition in human rotator cuff tendinopathy and tendinopathy in other anatomical regions, we aimed to evaluate perspectives of proteomics for progress in tendon tissue research. We expected that several proteins, including several MMPs, collagens, proteoglycans, and proinflammatory cytokines would be differentially expressed/represented in tendinopathy, and with regard to the rotator cuff, especially in case of tears. Molecular characterisation of protein profiles of different tendon disorders or stages of progression of tendon alterations may lead to a more thorough understanding of pathological pathways involved in tendon damage and thereby enable more efficient prevention, early diagnosis, and individualised treatment strategies.

## Materials and Methods

We conducted a systematic review in as close accordance with the PRISMA guidelines as possible, given the fact that the review does not evaluate healthcare interventions (the PRISMA checklist is included as S1 Appendix). We have not registered a protocol for the review.

### Literature search

A comprehensive, structured search was conducted in Medline, Embase, and Web of Science covering the period from 1 January 1990 to 18 December 2012. In Medline, we used the following search string: ((((matrix metalloproteinase* OR scleroprotein* OR cytokine* OR neuropeptide* OR glycoprotein* OR proteoglycan*) NOT medline[sb]) OR cytokines OR inflammation OR scleroproteins OR matrix metalloproteinases OR glycoproteins OR proteoglycans OR neuropeptides OR extracellular matrix proteins OR proteome OR proteomics OR RNA OR gene expression OR proteins) AND ((tendon injur* NOT tendon injuries[MeSH]) OR tendon injuries) OR ((tendinopathy OR ((tendino* OR tendini* OR tendon*) NOT medline[sb]))) OR (tendon AND (lacerations OR rupture)) OR rotator cuff tear OR Achilles tendon tear OR patellar tendon tear) AND humans) NOT review[sb]. Corresponding searches were performed in Embase and Web of Science (S2 Appendix). To focus specifically on proteomics research, we performed an additional specific search in Medline (S3 Appendix). Duplicates were removed and reference lists of retrieved articles were scanned for relevant articles missed by the original search.

### Study selection

The review was restricted to original articles in peer-reviewed journals, published in English, Danish, Norwegian, or Swedish. We selected human studies reporting on differential gene expression (mRNA) or protein composition in tendinopathy and/or tendon tears as compared to live or cadaveric controls using control tendon tissue from the same patient and joint (paired samples), healthy tendon tissue from other patients, but the same joint, or from different joints than the patient samples. In addition to studies, which examined gene expression and protein composition in rotator cuff or biceps tendinopathy, we included corresponding studies of Achilles, patellar, and posterior tibial tendinopathy because we expected the biological mechanisms involved in the pathogenesis of these disorders to be similar to those of rotator cuff and biceps tendinopathy[32, 33].

Studies using only cultivated cells, dialysate, or synovial, bursal, or capsular specimens were excluded, together with studies, which did not objectively quantify the outcomes. We also excluded studies, which did not use control tendon tissue. We excluded articles stepwise based firstly on title, secondly on abstract, and thirdly on full text.

### Data extraction

Papers were categorised according to their focus on transcripts (including transcriptomics), proteins (including proteomics), or both. We extracted information on first author, publication date, sample size, characteristics of study population, laboratory methods, number and names of transcripts/proteins searched for/identified, and direction of change. Data was extracted by the first author and crosschecked by a co-author (SWS or PF), and central information was presented in table form.

### Assessment of methodological quality

Methodological quality was assessed based on a set of 13 criteria modified from a checklist for assessing quantitative studies, where a single research question is not defined a priori (Table 1 and S4 Appendix)[34]. In our quality assessment, we rated studies that used paired samples from the same patient and joint or healthy tendon tissue from other patients, but the same joint, as controls higher than studies that used control tissue from different joints than the patient samples or from cadavers because there is a risk of bias due to regional anatomical differences or post-mortem changes. Total quality scores were calculated as percentages of the maximum possible scores. We considered a quality score >75 as indicative of good methodological quality.

**Table 1:** Quality criteria used to assess individual studies.

		Yes (2)	Partial (1)	No (0)	N/A	Comment
**1**	Was the question/objective sufficiently described?					
**2**	Was the study design and choice of experimental methods evident?					
**3**	Were selection and characteristics of patients and controls clearly described?					
**4**	Were patients and controls comparable on age and sex?					
**5**	Was the control tissue adequate?					
	Healthy sample or paired sample from same joint (Yes)					
	Cadaveric sample or sample from different joint (Partial)					
**6**	Was the sample size appropriate?					
**7**	Were the primary outcome measures evident and well-defined?					
**8**	Were the statistical methods described and justified?					
**9**	Was some estimate of variance reported for main results?					
**10**	Were results reported in sufficient detail?					
**11**	Were the results validated by use of other methods?					
**12**	Were the examiners blinded to disease state or other important characteristics?					
**13**	Were the conclusions supported by the results?					

Modified after Kmet, Lee, and Cook’s Standard Quality Assessment Criteria for evaluating primary research papers from a variety of fields[34]. Details are presented in S4 Appendix. N/A = not applicable.

The first author applied the checklist to all articles and consulted two co-authors (SWS and PF) in case of doubt (n = 3). Please note that the articles were only scored with regard to end points relevant to this paper.

### Assessment of publication bias

We plotted the direction of change of the most commonly examined MMP (MMP-3) against study size (number of patient samples), and visually inspected the plot for signs of publication bias. Inspired by the rationale behind funnel plots[35], the idea was that smaller studies would be more likely than larger studies to remain unpublished if the results pointed to a change in an unexpected direction.

## Results

**Fig 1:**
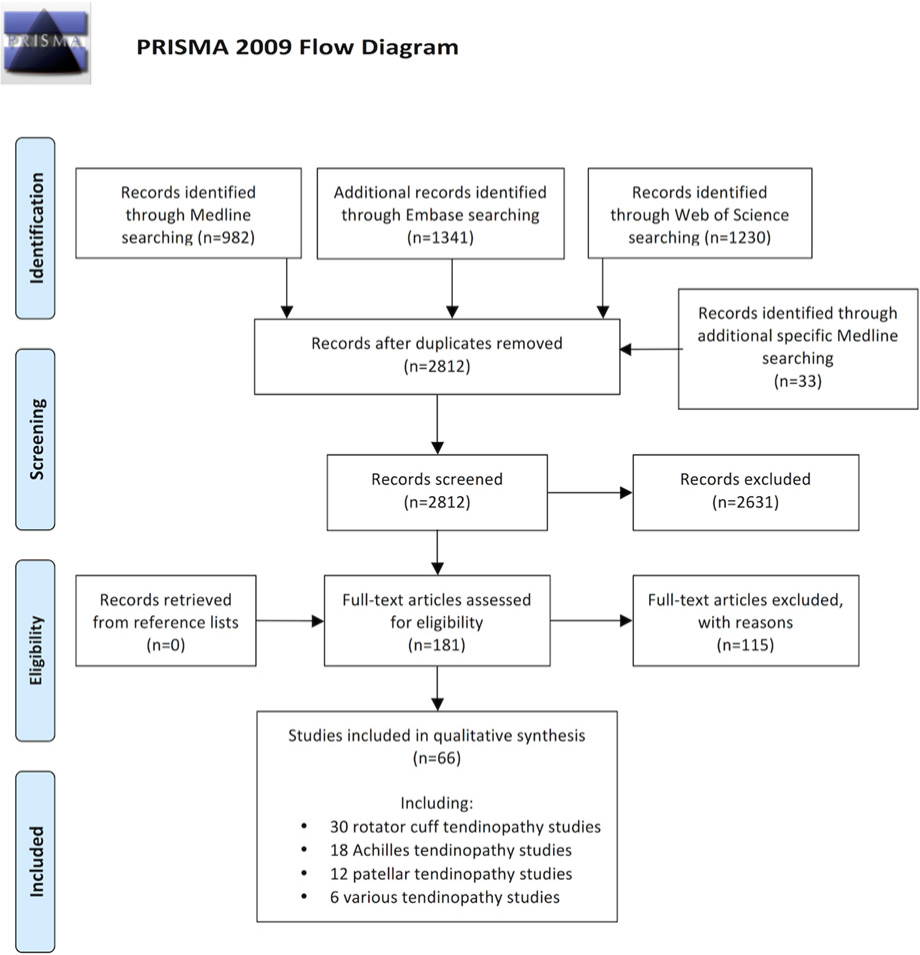
Flow diagram showing the inclusion process.

The primary searches yielded 2199 articles, of which we excluded 2097 based on title or abstract and 48 after full-text reading (a list of these 48 articles and reasons for their exclusion can be found in S5 Appendix). Of the 54 included articles, 25 dealt with rotator cuff or biceps tendinopathy[17, 18, 36–58], 14 with Achilles tendinopathy[33, 59–71], 3 with posterior tibial tendinopathy[72–74], 9 with patellar tendinopathy[75–83], 1 with both Achilles and patellar tendinopathy[84], 1 with both Achilles tendinopathy and posterior tibial tendinopathy[85], and 1 with pooled tendinopathic tissue from various anatomical locations[86]. Fig 1 displays the flow diagram of the inclusion process. Table 2 summarises characteristics and findings of rotator cuff and biceps studies. S1–S3 Tables show corresponding information regarding other tendons. In total, the 54 included articles comprised 975 specimens representing tendinopathy with or without tears and 508 control samples, and they explicitly evaluated the expression/representation of more than 140 prespecified transcripts and proteins. Table 3 shows experimental techniques used for the analysis of gene expression and protein composition. The predominant laboratory methods were real-time quantitative reverse transcriptase polymerase chain reaction (qRT-PCR) to detect and quantify mRNA, and Western blots and immunohistochemistry to detect and quantify proteins; only two studies used micro arrays.

**Table 2:** Gene expression and protein composition in rotator cuff tendinopathy and tears.

	Sample setting, anatomical site of sample, diagnosis, number of patients (n), mean age (range)			Direction of change of target tendon components				
First author, year	Patient samples	Control samples	Method	Up	Down	No difference	Comment	Quality
								Score
Bank, 1999[36]	Peroperative, ssp, degeneration, n = 10, n.r. (55–80)	Cadavers, ssp, normal, n = 39, n.r. (11–96)	Proteins*	-	Collagen	-	Results from crosslink and pentosidine (AGE) analysis are not included in this table	71
		Cadavers, bb, normal, n = 27, n.r. (11–96)						
Benson, 2009[37]	II: Peroperative, ssp, impingement, n = 9, n.r. (39–53)	I: Peroperative, ssc, normal from surgery for instability, n = 3, 19.7 (17–23)	Proteins	HIF-1α	-	-	Samples were divided into groups according to macroscopic appearance	69
	III: Peroperative, ssp, partial thickness tear, n = 3, 52.7 (40–67)	I		HIF-1α, BNip3	-	-	There was a positive correlation between apoptotic index and patient age, as well	
	IV: Peroperative, ssp, full thickness tear, n = 15, n.r. (17–69)	I		HIF-1α, BNip3	-	-	as the number of Bnip3 positive cells and patient age	
Chaudhury, 2011[38]	Peroperative, rc, tear, n = 92, 65.7 (45–89)	Peroperative, ssp, normal from other surgery, n = 11, 58 (46–79)	Proteins**	Elastin	Collagen I, Collagen II, Collagen III	Decorin	Samples were subdivided according to tear size	62
Hamada, 1997[39]	I: Peroperative, ssp, full thickness tear, n = 18, 56.7 (36–70)	III: Peroperative, ssp, normal from trauma surgery, n = 4, 41.8 (19–77)	Tran-scripts	α1-procollagen	-	-	Quantification based on number of label-positive cells in randomly selected areas	65
	II: Peroperative, partial thickness tear, n = 13, 54.6 (26–72)	III		α1-procollagen	-	-		
		I		-	-	α1-procollagen		
Joseph, 2009[40]	Peroperative lhb intraarticular portion, rotator cuff or biceps tendinopathy, n = 11, n.r. (36–60)	Peroperative, lhb extraarticular portion from same patient	Proteins	Collagen III, MMP-1, MMP-3	-	MMP-2, MMP-13, IGF	Correlation found between collagen type III and MMPs	83
Lakemeier, 2010[41]	Peroperative, ssp, full		Proteins				Quantification based on	77
Lakemeier, 2011[42]	thickness tear:						number of label-positive cells in relation to total number of cells	
	II: Patte grade 1, n = 6, 61 (55–68)	I: Peroperative, ssp, normal from trauma surgery, n = 6, 56 (47–69)		MMP-1, MMP-9, HIF-1α, VEGF	MMP-3	-	Patte classification refers to cuff tear retraction in the frontal plane:	
	III: Patte grade 2, n = 10, 65 (55–75)	I		MMP-1, MMP-9, HIF-1α, VEGF	MMP-3	-	1: Proximal stump close to bony insertion, 2: proximal	
		II		-	-	MMP-9	stump at the level of the	
	IV: Patte grade 3, n = 17, 69 (51–79)	I		MMP-1, MMP-9, HIF-1α, VEGF	MMP-3	-	humeral head, 3: proximal stump at glenoid level	
		II		MMP-9	-	-		
		III		-	-	MMP-9		
Lakemeier, 2010[43]	II: Peroperative, lhb, partial	I: Peroperative, lhb, normal	Proteins	MMP-1, MMP-9,	MMP-3	-	Quantification based on	77
Lakemeier, 2010[44]	thickness tear, n = 48, 61 (39–78)	from trauma surgery, n = 8, 56 (37–69)		VEGF			number of label-positive cells in relation to total number of cells	
	III: Peroperative, lhb, full thickness tear, n = 42, 67 (55–80)	I		MMP-1, MMP-9, VEGF	MMP-3	-		
		II, IV		-	-	MMP-1, MMP-3		
	IV: Peroperative, lhb, cuff arthropathy, n = 18, 70 (51–87)	I		MMP-1, MMP-9, VEGF	MMP-3	-		
		II, III		-	-	MMP-1, MMP-3, VEGF		
Lo, 2004[45]	Peroperative, rc, full thickness tear, n = 10, 59.2±4.4 (n.r.)	Cadavers, rc, normal, n = 6, 74±7 (n.r.)	Tran-scripts	MMP-13	MMP-3, TIMP-2, TIMP-3, TIMP-4	MMP-1, MMP-8, MMP-10, TIMP-1	RT-PCR normalised to GADPH	83
			Proteins	MMP-13	-	-		
Lo, 2005[46]	Peroperative, rc, full thickness tear, n = 10, 57.5±7.3 (n.r.)	Cadavers, rc, normal, n = 6, 74±7 (n.r.)	Tran-scripts	Collagen I, Collagen III, aggrecan	Decorin	Collagen II, biglycan	Results on bursal tissue are not included in this table. RT-PCR normalised to GADPH	79
Lundgreen, 2011[47]	Peroperative, ssp, full thickness tear, n = 15, 57.7 (49–69)	Peroperative, ssc, normal from arthroscopic labral repair, n = 10, 43.9 (32–51)	Tran-scripts	-	HDAC1, MDM4, PPM1D, NF-κβ	-	Gene expression analyses by RT-PCR were performed on a subset of the torn ssp and reference ssc	73
			Proteins	p53, ki67	-	-	RT-PCR normalisation gene n.r.	
Millar, 2008[18]	I: Peroperative, ssp tear,	Ia: Peroperative, ssc, normal	Tran-	MIF, IL-18, IL-	-	-	Results from a rat study are	85
Millar, 2009[48]	n = 17, 57 (39–76)	from same patient	scripts	15, IL-6, Cap-3, Cap-8, HSP-70, HSP-27			not included in this table. RT-PCR normalised to β-actin	
	I	II: Peroperative, ssc, normal from surgery for instability, n = 10, 35 (20–41)	Tran-scripts	MIF, IL-18, IL-15, IL-6, Cap-3, Cap-8, HSP-70, HSP-27	TNFα, cFlip	-		
	I, Ia	II	Tran-scripts	MIF, IL-18, IL-15, IL-6, Cap-3, Cap-8, HSP-70, HSP-27	-	-		
	I, Ia	II	Proteins	MIF, IL-18, IL-15, IL-6, Cap-3, Cap-8, HSP-70, HSP-27, TNFα	cFlip	-		
Millar, 2012[17]	I: Peroperative, ssp tear, n = 15, 55 (38–70)	Ia: Peroperative, ssc, normal from the same patient	Proteins	-	HIF-1α, clusterin, Bcl-2	VEGF	Results from an in vitro hypoxia study are not included in this table	81
		II: Peroperative, ssc, normal from surgery for instabililty,n = 10, 32 (17–38)						
	I, II	Ia	Proteins	-	HIF-1α, clusterin, Bcl-2	VEGF		
Oliva, 2009[49]	Peroperative, ssp tear, n = 5, 60±1 (n.r.)	Cadaver, ssp, normal, n = 5, 65±1 (n.r.)	Tran-scripts	-	TG2	TG1, FXIII	Results from a mouse study are not included in this table	54
			Proteins	FXIII	TG2	-	RT-PCR normalisation gene n.r.	
Riley, 1994[50]	II: Peroperative, ssp tear, n = 26, 59.6 (38–80)	I: Cadaver, ssp, normal, n = 60, 57.7 (11–95)	Proteins	Collagen III	(Total collagen)	-		75
	III: Peroperative, ssc tear, n = 8, 73.4 (68–80)	Ia: Cadaver, common biceps, n = 24, 53.6. (12–83)						
Riley, 2002[51]	Peroperative, rc, partial/ full thickness tear, n = 10, n. r. (55–80)	I: Cadaver, biceps, normal, n = 24, n. r. (18–99)	Proteins	MMP-1	-	MMP-2, MMP-3	Number of samples per patient/cadaver not specified	79
		II: Cadaver, ssp, normal, n = 29, n. r. (18–96)		MMP-1, denatured collagen	MMP-2, MMP-3	-		
Qi, 2012[58]	Peroperative, biceps, rotator cuff repair, n = 11, n.r. (27–67)	Peroperative, flexor radialis carpi, normal, n = 5, n.r. (27–67)	Tran-scrips	Tenomodulin isoform II	-	Tenomodulin isoform I, tenomodulin isoform III	qPCR normalised to 18s rRNA. Results from cell studies are not included in this table	86
Shindle, 2011[52]	Ia: Peroperative, ssp, full thickness tear, n = 24, 62.4±2.0 (n.r.)	IIa: Peroperative, ssp, partial thickness tear, n = 16, 56.3±1.7 (n.r.)	Tran-scripts	MMP-9, MMP-13, COX-2, (COL1A1)	iNOS	COL3A1, Il-1β, IL-6, TNFα, VEGF, MMP-1, TIMP-1, SMA, biglycan	Results on synovial and bursal specimens are not included in this table	92
		IIb: Peroperative, ssc, normal from same patient		-	(VEGF), COL3A1, biglycan	MMP-1, MMP-9, MMP-13, COX-2, iNOS, Il-1β, IL-6, TNFα, TIMP-1, SMA	RT-PCR normalised to GADPH	
Shirachi, 2011achi[53]	Peroperative, rc, full thickness tear, n = 12, 58.2 (47–68)	Cadaver, rc, normal, n = 5, 66.2 (57–76)	Tran-scripts	Collagen I, Collagen III	-	-	Only 5 tear samples and 2 control samples were subjected to protein analysis	75
			Proteins	Collagen I, Collagen III	-	-	RT-PCR normalised to β-actin	
Singaraju, 2008[54]	Peroperative, lhb, biceps tendinopathy, n = 6, 51 (44–60)	Cadaver, lhb, normal, n = 6, 76 (42–81)	Proteins	-	-	CGRP, SP	Relative intensities of SP and CGRP were determined through subjective scoring	31
Tillander, 2002[55]	II: Peroperative, ssp, impingement, n = 16, 51 (30–61)	I: Peroperative, ssc, normal from instability patients, n = 9, 28 (20–37)	Proteins	Fibronectin, (MMP-1)	-	-	MMP-1 was only found in few patients in groups II and III	69
	III: Peroperative, ssp tear, n = 7, 57 (41–73)	I		(MMP-1)	Fibronectin	-		
Tomonaga, 2000[56]	Peroperative, ssp, full thickness tear, n = 28, 55.9 (36–77) and ssp, partial thickness tear, n = 14, 54.6 (26–72)	I: Peroperative, ssp normal (other surgery), n = 4, 40.8 (19–73)	Tran-scripts				Results on synovial specimens are not included in this table	46
	II: Apparent trauma: 25 of the above 42 patients	I		Procollagen α1 type III	-	-		
	III: No apparent trauma: 17 of the above 42 patients	I		-	Procollagen α1 type III	-		
Wang, 2001[57]	Peroperative, degenerative ssp, n = 13(?), n.r. (n.r.)	Peroperative, ssc, normal from same patient	Tran-scripts	PRDX5	-	-	RT-PCR normalised to β-actin	65
			Proteins	PRDX5	-	-	Number of patients included is not clearly stated in the article	

Studies examining biceps tendon samples are included in this table. None of the studies that quantified proteins used proteomics technologies. Two authors in same row indicate that the same patient and control populations were used in the two studies; () = non-significant trend.

Abbreviations: bb = biceps brachii tendon, bcl = B cell lymphoma, cap = caspase, CB_1_R = cannabinoid receptor type 1, CCL = chemokine ligand, CGRP = calcitonin gene related protein, COX = cyclooxygenase, FXIII = factor XIII, GADPH = glyceraldehyde 3-phosphate, HDAC = histone deacetylase, HIF-1α = hypoxia inducible factor-1α, HSP = heat shock protein, IGF = insulin-like growth factor, IL = interleukin, iNOS = inducible nitric oxide synthase, lhb = long head of biceps tendon, MDM4 = double minute 4 protein, MIF = macrophage migration inhibitory factor, MMP = matrix metalloproteinase, NFκB = nuclear factor kappa-light-chain-enhancer of activated B cells, n.r. = not reported, p53 = protein 53, PPM1D = protein phosphatase 1D, PRDX = peroxiredoxin, rc = rotator cuff tendon, SMA = smooth muscle actin, SP = substance P, ssc = subscapularis tendon, ssp = supraspinatus tendon, TG = transglutaminase, TIMP = tissue inhibitor of metalloproteinases, TNF-α = tumor necrosis factor-α, VEGF = vascular endothelial growth factor.

* Reversed-phase high performance liquid chromatography.

** Fourier transform spectroscopy.

**Table 3:** Experimental techniques used for the analysis of gene expression and protein composition.

mRNA	Protein
RT-PCR	Western blot
Micro arrays	ELISA
Northern blot	Immunohistochemistry
RNA-seq	Radioimmunoassays
	Mass spectrometry

Abbreviations: RT-PCR = reverse transcription polymerase chain reaction, RNA-seq = RNA sequencing, ELISA = enzyme-linked immunosorbent assay.

Studies were fairly evenly distributed across study types; about a third focused on gene expression (mRNA), a third on protein representation, and a third on both gene expression and protein representation. In our additional specific Medline search, we identified 13 proteomics studies, of which 5[87–91] had not been captured in the comprehensive searches. Altogether we identified 14 studies that applied proteomics technologies on tendon tissues/cells, but none of them were eligible for inclusion in the present review: 1 study was only presented as a conference abstract[92], 10 were conducted on animal tendons or cultured cells[88, 90, 91, 93–99], and 3 were conducted on human tendons to examine exercise-induced changes[100], drug concentrations[101], or laboratory procedures[102].

### Quality assessment

Quality scores are listed in Table 2 and S1–S3 Tables. Details are presented in S6 Appendix. No study achieved the maximum quality score; however 56% scored >75. Major reasons for lower scores were poor comparability between tendinopathy/tear samples and control samples, primarily with respect to age, a scarcity of healthy tendon control samples especially in studies of rotator cuff tendons, lacking validation of results against other methods, and blinding of examiners to disease state. Furthermore, many studies failed to specify inclusion criteria.

### Surgical specimens and control tissue

Studies of rotator cuff tendinopathy relied predominantly on surgical specimens from degenerated or torn supraspinatus tendons compared with ipsilateral subscapularis control specimens or cadaveric supraspinatus controls (Table 2). Patients with patellar tendinopathy were often in their 30s, while patients with Achilles tendinopathy tended to be in their 40s and patients with rotator cuff tendinopathy in their 50s and 60s (Table 2 and S1–S3) Tables. Across anatomical locations, cadaveric controls tended to span a wider age range and were often older than the patients. Both men and women were included in 71% of the studies, but relatively few considered potential sex-related differences[45, 46, 72, 73, 84–86, 103].

### Differential gene expression and protein representation

Across anatomical locations, the majority of studies (40 of 54 studies) focused on collagens, MMPs, TIMPs, and/or proinflammatory cytokines. The content of the following proteins tended to be increased in tendinopathy: aggrecan (3 positive studies out of 4), fibronectin (3 of 3 studies), tenascin C (TNC) (2 of 3 studies), cyclooxygenase (COX)-2 (3 of 3 studies), collagen I (11 of 15 studies) and III (13 of 14 studies), MMP-1 (6 of 12 studies), -9 (7 of 7 studies), and -13 (4 of 7 studies), TIMP-1 (4 of 7 studies), and vascular endothelial growth factor (VEGF) (4 of 7 studies). For MMP-2, 5 of 11 studies found increased levels relative to controls, and for biglycan and versican, this was the case for 2 of 6 studies and 3 of 8 studies, respectively; the remainder did not observe any difference, except that one study found decreased levels of three versican variants in painful and ruptured Achilles tendons[61]. Meanwhile, the content of MMP-3 (10 of 12 studies) tended to be decreased, and there were some indications that this was also the case for TIMP-2 (2 of 6 studies), -3 (2 of 5 studies), and -4 (2 of 4 studies). Results with respect to a disintegrin and metalloproteinase with thrombospondin motifs (ADAMTS), a disintegrin and metalloproteinase (ADAM), and proinflammatory cytokines were inconsistent, or few studies reported their regulation/expression. The direction of change of the most frequently examined transcripts and proteins was similar across tendon type. This was particularly evident for collagen I and-III, MMP-3, -9, and -13, and VEGF, which were examined in all four tendon groups. In general, each study reported on only a few selected transcripts and/or proteins.

Particularly with regard to the rotator cuff, there was a high degree of heterogeneity between studies with respect to the examined transcripts and proteins, and few studies compared specimens from degenerated and torn supraspinatus tendons. In one study, levels of BNip3 positive cells were higher in samples from patients with tears compared with samples from patients with subacromial impingement syndrome, combined with a rise in ‘apoptotic index’[104]. This might indicate that the pathological features of subacromial impingement syndrome are exacerbated in tears, in support of the theory of progressive failure of the rotator cuff. A group of similar studies[42–44], in which supraspinatus or long head of biceps tendon samples from torn rotator cuffs were divided in groups according to tear size, indicated that the expression/representation of HIF-1α[43], VEGF[43], and MMP-9[41, 42] was enhanced as rotator cuff pathology worsened. Two studies found similar correlations between increasing expression/representation of VEGF[44], MMP-1, and -9[41] and increasing extent of a full-thickness rotator cuff tear, but these findings were not statistically significant. On the other hand, two studies of apoptosis and cytokines in torn supraspinatus tendons showed no correlation between apoptotic[18]and cytokine gene expression[48] and tear size/histological grade, and a similar study even found an inverse correlation between tear size and apoptotic markers[17].

### Publication bias

**Fig 2:**
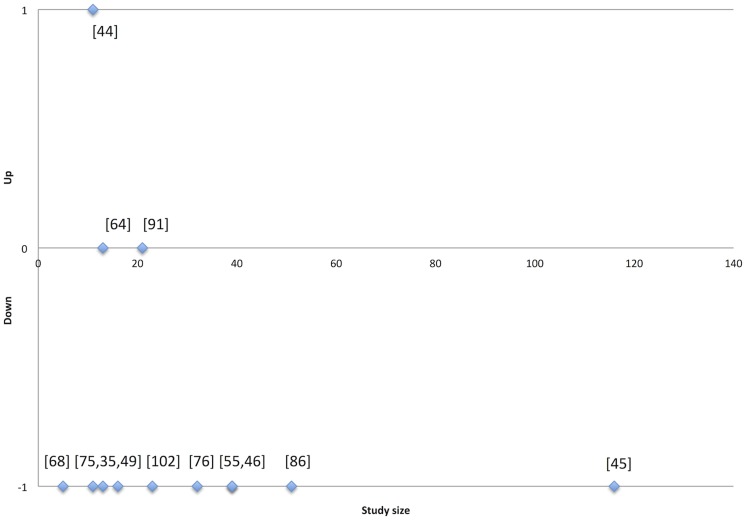
Direction of change of matrix metalloproteinase 3 (MMP-3) in relation to study size (number of patient samples). Each dot marks the direction of change in MMP-3 in a single study[33,40–42,45,50,59,66,67,73,78,86].

To examine the possibility of publication bias, we plotted the direction of change of MMP-3 content in relation to study size (Fig 2). Since the content of MMP-3 tended to be decreased in tendinopathy, the plot would indicate publication bias if smaller studies tended to report a decrease of MMP-3 in tendinopathy less often than larger studies did. Study size varied from 5 to 116 patient samples; only two studies had a size of >50. Two studies reported both an increase and a decrease in MMP-3 for different comparisons; in these cases, we chose to report only the result from the comparison between patient and control samples[41] and prioritised samples taken from the same anatomical location[51]. One study showed only a borderline decrease in MMP-3[78], but we presented the result as a decrease in the figure. Five studies had a sample size ≤20, of which four reported a lower expression/representation of MMP-3 (80%). Seven studies had sample a size >20, of which six reported a lower expression/representation of MMP-3 (86%). Thus, similar results were reported in small and large studies.

## Discussion

This systematic review was performed to examine the literature on differential gene expression and protein composition in human tendinopathy including tears, as well as to provide insights into the potential of proteomics. Across anatomical regions, several gene transcripts and proteins were differentially expressed/represented in samples from patients with tendinopathy compared to control samples. The most consistent findings were an increase in collagens I and III, MMP-1, -9, -13, TIMP-1, and VEGF, and a decrease in MMP-3. With regard to the rotator cuff, it was not possible to determine, whether specific changes were characteristic of tears as compared with tendinopathy. Our most important result was that we were unable to identify any proteomics studies that fulfilled our inclusion criteria.

Proteomics has gained interest in studies of other musculoskeletal disorders[27–31]. To search specifically for proteomics studies on musculoskeletal topics, we developed an additional specific search string, which retrieved these other studies (S3 Appendix). Using this string, we identified five studies, which had not already been captured by the comprehensive searches, but still none that fulfilled our inclusion criteria. A recent review (which we return to below) did not include studies that we missed[105]. Thus, we find it unlikely that we missed important proteomics studies of tendinopathy. Publication bias and bias due to selective reporting of findings within a study may have influenced our results. Our plot of the findings for MMP-3 in relation to sample size (Fig 2) was not a very sensitive method of detection since almost all the depicted studies were small. Nonetheless reassuringly, we did not reveal any indication of such bias.

The transition from healthy tendon to tendinopathy must be gradual and it is difficult to draw a clear line between pathological and control specimens. Control specimens in terms of bursal and subscapularis tissues as well as the intraarticular portion of the long head of the biceps tendon from joints with supraspinatus tendinopathy including tears often showed signs of degeneration[40, 52, 106, 107], which suggests a global affection of different subacromial soft tissues. For example, degenerative changes were found in subscapularis tendons from patients with supraspinatus tears, even though they appeared normal on preoperative magnetic resonance imaging[52]. Using paired subscapularis controls to analyse differential protein composition may therefore underestimate actual differences from normal. Furthermore, the use of subscapularis controls or tendons from other anatomical locations may add bias due to normal, functional variations in tendon composition[108–111]. Strictly standardised biopsy-procedures are probably important to ensure reproducibility and account for regional variations in protein composition within a tendon. Moreover, human tendon specimens often represent late stages of disease, and unless time-sequential tissue samples are obtained, it is impossible to determine, whether observed changes preceded or succeeded a rupture. The use of cadaveric controls also entails problems; post-mortem changes may interfere with the results and, according to our findings, the cadaveric controls were often older or spanned a wider age range than the patients. The prevalence of rotator cuff tendinopathy increases with age[112, 113] and studies of cadaveric rotator cuff tendons have revealed that the proportion of samples showing histological signs of degeneration increases with age even in undiagnosed cadavers[114, 115]. As a minimum, cadaveric controls should therefore be comparable to the patients with respect to age.

In the following, we interpret our results in view of contributory evidence from animal and cell culture studies as well as other research areas. We also discuss our findings and interpretations against a recent review of histological and molecular changes in rotator cuff disease[105]. Apart from methodological differences regarding searching and critical appraisal of the literature, the just-mentioned review differed from ours in that it was restricted to rotator cuff tendons, included animal and in vitro studies, and did not focus on proteomics research. Based on an outline of general shortcomings in studies of transcripts and proteins using probes, primers, and antibodies, we then evaluate the potential of proteomics in tendon research.

### Collagens

An increased expression of collagen III was related to tendinopathy including tears in all[40, 46, 53, 64, 66, 69, 70, 73, 74, 79, 86, 116] but 2[33, 38] of 45 included studies, which examined this protein. This change was evident both with respect to gene expression, i.e. transcripts (mRNA), and with respect to protein representation. The ratio of collagen I to collagen III was often decreased in tendinopathic specimens. A decreased ratio of collagen I to collagen III may be interpreted as a sign of tissue remodelling[110] and has previously been associated with decreased mechanical stability and a propensity for inguinal hernia recurrence after surgical repair[117]. Collagen III is normally synthesised in early stages of wound healing and is considered to be an immature form of collagen I[110, 118]. Collagen III fibrils have a thinner diameter, and are more elastic and less organised than collagen I.

In healthy tendons, advanced glycation end products (AGEs) are formed in an irreversible reaction and these products therefore tend to accumulate in long-lived proteins like collagen[36, 119, 120]. After 50 years of age, even normal supraspinatus tissue shows signs of tissue remodelling in the form of decreased levels of pentosidine (a non-protein biomarker for AGEs—low levels of pentosidine indicate high contents of AGEs). In tendinopathic and ruptured tendons this remodelling process appears to be accelerated[33, 36, 72]. As a result, pathological supraspinatus tendons appear to be biologically younger than healthy supraspinatus tendons[36]. This may indicate an up regulated tissue turnover in tendinopathy, consistent with the increase in collagen III. The recent review[105] also suggested this theory of an up regulated tissue turnover in degenerated tendons resulting in formation of a mechanically less stable matrix.

### Matrix metalloproteinases

MMPs have been implicated in the pathogenesis of rotator cuff tendinopathy[5, 105, 121]. MMPs are able to degrade all kinds of ECM proteins, function as regulators of ECM homeostasis, and are key players in tendon healing and remodelling[5, 121]. They may be involved in the mobilisation of growth factors and cytokines through cleavage of cytokine-binding proteins[122]. According to our review, an increase of MMP-1 (collagenase-1), -9 (gelatinase B), and -13 (collagenase-2), and a decrease in MMP-3 (stromelysin-1) were frequent findings in pathological tendon specimens, along with a tendency towards an increase of MMP-2 (gelatinase A). Similar results were reported in the recent review[105]. This might indicate a shift in metabolism towards matrix degradation, which corresponds well with the observed decrease in the ratio of collagen I to collagen III, and decreased pentosidine levels. MMP-3 is a proteolytic enzyme believed to have regulatory functions in the ECM [123–125] and it is thought to play a part in the regulation of connective tissue remodelling, as it acts as an activator of other MMPs[5]. Interestingly, polymorphisms within the MMP-3 gene have been associated with anterior cruciate ligament ruptures[16] and Achilles tendinopathy[15].

### Proteoglycans and glycoproteins

There is a tendency towards an increase in proteoglycans as well as a well-established increase in glycosaminoglycan (GAG) (polysaccharide) content in tendinopathy[73, 126]. This tendency towards a fibrocartilaginous tissue type characterised by increases in the large proteoglycans biglycan and aggrecan and a decrease in decorin was also observed in the recent review[105]. GAGs are able to bind water molecules, and this may account for tendon swelling in tendinopathy. Increases in tendon proteoglycans are often interpreted as a response to compressive loading[62, 127, 128]. Thus, differences in the mechanical environment at the site of biopsy may in part explain the differences in results that we observed between studies. TNC is an elastic glycoprotein, which is often up regulated in response to compressive[129, 130] or tensional[131] loading. Furthermore, TNC is believed to be involved healing processes as well as in the tendon’s adaptation to mechanical stress[129, 132], possibly by providing elasticity to the tendinous tissues[133, 134]. In vitro studies suggest that TNC may also induce inflammatory mediators and matrix degradation in cartilage from osteoarthritic joints[135].

### Inflammation and degeneration

In recent reviews, it has been suggested that inflammation and degeneration in tendinopathy are not mutually exclusive, but are instead closely interconnected[1, 21, 136, 137]. The inflammatory theory has often been disregarded due to a lack of evidence of inflammatory cell infiltrates in human tendinopathic specimens, but recently, cytokines related to inflammation have been found[4, 48, 52, 65, 85]. Cytokines are able to influence a wide array of ECM components. In vitro studies of human tenocytes have suggested that tendon contents of several cytokines and neuropeptides may vary in a loading-dependent manner[105, 138–141]. Mediators of neurogenic inflammation—i.e., inflammation arising from the release of neuropeptides from afferent neurons[142]—including substance P[143, 144], calcitonin gene related peptide (CGRP)[145], glutamate[81], and the proangiogenetic cytokine, VEGF[43, 44, 71, 82], have also been associated with tendinopathy. Inflammation may persist in the early stages of tendinopathy and initiate a cascade of catabolic events in the tendon. In fact, a close-knit relationship seems to exist between several pro-inflammatory cytokines and MMPs; for example, in vitro studies have recently shown that interleukin(IL)-1β can induce COX-2, MMP-1, -9, and -13, ADAMTS-4, IL-6 and IL-1β in human tendon cells[146], and a strong correlation has been found between the inflammatory cytokine IL-1β and MMP-9[52]. Several cytokines and growth factors play an important role in tendon healing[147–149], and degenerated and ruptured supraspinatus tendons share many biochemical characteristics of wound healing[118]. Cytokines and growth factors are normally increased during wound healing and as mentioned above several may act as proinflammatory mediators. An increase in numerous cytokines and growth factors was also found in the recent review, and the up regulation of proinflammatory cytokines was suggested to result either from ongoing healing or from an imbalance between anabolic and catabolic tendon processes[105]. In our review, this was reflected by differential expression/representation of various matrix-degrading enzymes (MMPs), collagens, and other structural matrix proteins.

### Rotator cuff tendinopathy versus other tendinopathies

We included studies on tendons from various anatomical locations under the assumption that the biological processes leading to tendon degeneration are similar in different tendons. On the other hand, tendons from different anatomical locations vary with respect to morphological and mechanical properties depending on their specialised function, and hence, the pathways to degeneration may vary as well. For example, a distinction is often made between spring-like tendons that are subjected to high strain, and positional tendons that are subjected to lower strain[150].

### Transition from rotator cuff tendinopathy to development of tears

It has long been assumed that there exists a continuum between rotator cuff tendinopathy and the development of tears[151], and we would expect this to be reflected in the protein profiles of samples from patients representing these conditions. Few of the included studies compared rotator cuff tendinopathy and rotator cuff tears. Several of these studies indicated that changes in tendon gene expression and protein representation were exacerbated with worsening pathology, but findings were inconsistent. It is possible that only certain types of proteins show a pattern of worsening with cuff pathology, or, indeed—as one study of apoptosis in rotator cuff tears suggested[17]—that degenerative and inflammatory activity of tendinopathic tendons decreases following rupture.

### Proteomics in tendon tissue research

Since proteins are functional effectors of most cellular functions, the study of proteins may provide insights into disease processes, including those that are not reflected on an mRNA level (as mentioned in the introduction, there is no direct relationship between mRNA expression and protein levels). Previously, proteomics technologies required relatively large amounts of tissue, and this may have prevented proteomics studies on patient tendon samples. In recent years, however, novel MS technologies have allowed reliable analyses of low milligram amounts of biopsy material[152].

Proteomics is primarily based on the use of MS, a highly sensitive analytical tool that uses electrical fields to measure the masses of charged molecules[25, 153–156]. However, high-abundance proteins may mask low-abundance proteins. The high amounts of collagen I in tendon tissue pose a significant challenge and collagen depletion prior to analysis may be necessary. Although MS-based proteomics studies may fail to uncover low-abundant proteins, they may provide comprehensive analyses of the overall protein composition in different stages of tendinopathy.

Proteomics technologies offer new ways of looking at diseases and how they progress. In a single specimen, proteomics analyses may identify hundreds of proteins, and so, a major challenge in MS-based proteomics is to sort out important information from overwhelming amounts of data. This is accomplished with help of bioinformatics, an interdisciplinary field that comprises a range of computational tools (e.g. sequence databases and search algorithms) used to analyse biological data and locate proteins and their biological functions.

The field of proteomics is constantly evolving, and use of these techniques to characterise and quantify differences in protein composition in tendinopathic and healthy samples may improve our understanding of biological processes leading to tendinopathy and tendon tears.

## Conclusions

In studies based on methods, which only allowed simultaneous quantification of a limited number of prespecified mRNA molecules or proteins, this review found several markers of tissue remodelling to be differentially expressed/represented in human tendinopathy, most notably collagen I and III, MMP-1, -3, -9, and -13, TIMP-1, and VEGF. With regard to the rotator cuff, it was not possible to determine, whether specific changes were characteristic of tears as compared with tendinopathy. We were unable to identify any proteomics studies of tendinopathic or torn tendon samples from humans, which fulfilled our inclusion criteria. Proteomics technologies may be a way to identify protein profiles (including unexpected proteins) that characterise specific tendon disorders or stages of tendinopathy, and thereby enhance our understanding of pathways involved in tendon damage. Thus, our results suggested an untapped potential for proteomics in tendon research.

## Supporting Information

S1 Fig OMICS technologies. The complexity and size of each compartment increase with each step down the pyramid. Adapted from Holmes et al[26].
(TIFF)

S1 Table Gene expression and protein composition in patellar tendinopathy. None of the studies that quantified proteins used proteomics technologies. Two authors in same row indicate that the same patient and control populations were used in the two studies; () = non-significant trend.
(DOCX)

S2 Table Gene expression and protein composition in Achilles tendinopathy and ruptures. None of the studies that quantified proteins used proteomics technologies. Two authors in same row indicate that the same patient and control populations were used in the two studies; () = non-significant trend, [] = could not be detected in all samples.
(DOCX)

S3 Table Gene expression and protein composition in various tendinopathies. None of the studies that quantified proteins used proteomics technologies. Two authors in same row indicate that the same patient and control populations were used in the two studies; () = non-significant trend.
(DOCX)

S1 Appendix PRISMA Checklist. (DOC)

S2 Appendix Search strings used in Embase and Web of Science. (DOCX)

S3 Appendix Additional specific search string used for specific proteomics search in Medline. (DOCX)

S4 Appendix Manual for quality scoring in Table 1 adapted from Kmet, Lee, and Cook’s Standard Quality Assessment Criteria for evaluating primary research papers from a variety of fields [34]. (DOCX)

S5 Appendix Reasons for exclusion after full-text reading (n = 48). Information from some of the excluded papers is used in the discussion.
(DOCX)

S6 Appendix Details on quality scoring of included studies. N/A = not applicable.
(DOCX)

## References

[1] Fredberg U, Stengaard-Pedersen K. Chronic tendinopathy tissue pathology, pain mechanisms, and etiology with a special focus on inflammation. Scand J Med Sci Sports. 2008;18: 3–15. 10.1111/j.1600-0838.2007.00746.x 18294189

[2] Chaudhury S, Carr AJ. Lessons we can learn from gene expression patterns in rotator cuff tears and tendinopathies. J Shoulder Elbow Surg. 2012;21: 191–199. 10.1016/j.jse.2011.10.022 22244062

[3] Riley GP. Gene expression and matrix turnover in overused and damaged tendons. Scand J Med Sci Sports. 2005;15: 241–251. 15998341 10.1111/j.1600-0838.2005.00456.x

[4] Bedi A, Maak T, Walsh C, Rodeo SA, Grande D, Dines DM, et al Cytokines in rotator cuff degeneration and repair. J Shoulder Elbow Surg. 2012;21: 218–227. 10.1016/j.jse.2011.09.020 22244065

[5] Del Buono A, Oliva F, Longo UG, Rodeo SA, Orchard J, Denaro V, et al Metalloproteases and rotator cuff disease. J Shoulder Elbow Surg. 2012;21: 200–208. 10.1016/j.jse.2011.10.020 22244063

[6] Miranda H, Viikari-Juntura E, Heistaro S, Heliovaara M, Riihimaki H. A population study on differences in the determinants of a specific shoulder disorder versus nonspecific shoulder pain without clinical findings. Am J Epidemiol. 2005;161: 847–855. 15840617 10.1093/aje/kwi112

[7] Roquelaure Y, Bodin J, Ha C, Petit Le Manac'h A, Descatha A, Chastang JF, et al Personal, biomechanical, and psychosocial risk factors for rotator cuff syndrome in a working population. Scand J Work Environ Health. 2011;37: 502–511. 10.5271/sjweh.3179 21706122

[8] Shiri R, Varonen H, Heliovaara M, Viikari-Juntura E. Hand dominance in upper extremity musculoskeletal disorders. J Rheumatol. 2007;34: 1076–1082. 17343320

[9] Vitale MA, Arons RR, Hurwitz S, Ahmad CS, Levine WN. The rising incidence of acromioplasty. J Bone Joint Surg Am. 2010;92: 1842–1850. 10.2106/JBJS.I.01003 20686058

[10] Nordqvist A, Rahme H, Hovelius L, Etzner M. [Shoulder diseases]. Lakartidningen. 2007;104: 1492–1496. 17550025

[11] Svendsen SW, Frost P, Jensen LD. Time trends in surgery for non-traumatic shoulder disorders and postoperative risk of permanent work disability: a nationwide cohort study. Scand J Rheumatol. 2012;41: 59–65. 10.3109/03009742.2011.595375 22103333

[12] Seitz AL, McClure PW, Finucane S, Boardman ND 3rd, Michener LA. Mechanisms of rotator cuff tendinopathy: intrinsic, extrinsic, or both? Clin Biomech. 2011;26: 1–12. 10.1016/j.clinbiomech.2010.08.001 20846766

[13] Mokone GG, Gajjar M, September AV, Schwellnus MP, Greenberg J, Noakes TD, et al The guanine-thymine dinucleotide repeat polymorphism within the tenascin-C gene is associated with achilles tendon injuries. Am J Sports Med. 2005;33: 1016–1021. 15983124 10.1177/0363546504271986

[14] Mokone GG, Schwellnus MP, Noakes TD, Collins M. The COL5A1 gene and Achilles tendon pathology. Scand J Med Sci Sports. 2006;16: 19–26. 16430677 10.1111/j.1600-0838.2005.00439.x

[15] Raleigh SM, Van Der Merwe L, Ribbans WJ, Smith RKW, Schwellnus MP, Collins M. Variants within the MMP3 gene are associated with Achilles tendinopathy: Possible interaction with the COL5A1 gene. Br J Sports Med. 2009;43: 514–520. 10.1136/bjsm.2008.053892 19042922

[16] Malila S, Yuktanandana P, Saowaprut S, Jiamjarasrangsi W, Honsawek S. Association between matrix metalloproteinase-3 polymorphism and anterior cruciate ligament ruptures. Genet Mol Res. 2011;10: 4158–4165. 10.4238/2011.October.31.1 22057989

[17] Millar NL, Reilly JH, Kerr SC, Campbell AL, Little KJ, Leach WJ, et al Hypoxia: a critical regulator of early human tendinopathy. Ann Rheum Dis. 2012;71: 302–310. 10.1136/ard.2011.154229 21972243

[18] Millar NL, Wei AQ, Molloy TJ, Bonar F, Murrell GA. Heat shock protein and apoptosis in supraspinatus tendinopathy. Clin Orthop Relat Res. 2008;466: 1569–1576. 10.1007/s11999-008-0265-9 18459030 PMC2505259

[19] Yuan J, Wang MX, Murrell GA. Cell death and tendinopathy. Clin Sports Med. 2003;22: 693–701. 14560541 10.1016/s0278-5919(03)00049-8

[20] Wren TA, Lindsey DP, Beaupre GS, Carter DR. Effects of creep and cyclic loading on the mechanical properties and failure of human Achilles tendons. Ann Biomed Eng. 2003;31: 710–717. 12797621 10.1114/1.1569267

[21] Abate M, Silbernagel KG, Siljeholm C, Di Iorio A, De Amicis D, Salini V, et al Pathogenesis of tendinopathies: inflammation or degeneration? Arthritis Res Ther. 2009;11: 30. 10.1186/ar2723 PMC2714139 19591655

[22] van Rijn RM, Huisstede BM, Koes BW, Burdorf A. Associations between work-related factors and specific disorders of the shoulder—a systematic review of the literature. Scand J Work Environ Health. 2010;36: 189–201. 20094690 10.5271/sjweh.2895

[23] Svendsen SW, Bonde JP, Mathiassen SE, Stengaard-Pedersen K, Frich LH. Work related shoulder disorders: quantitative exposure-response relations with reference to arm posture. Occup Environ Med. 2004;61: 844–853. 15377771 10.1136/oem.2003.010637 PMC1740658

[24] Vlaanderen J, Moore LE, Smith MT, Lan Q, Zhang L, Skibola CF, et al Application of OMICS technologies in occupational and environmental health research; current status and projections. Occup Environ Med. 2010;67: 136–143. 10.1136/oem.2008.042788 19933307 PMC2910417

[25] de Hoog CL, Mann M. Proteomics. Annu Rev Genomics Hum Genet. 2004;5: 267–293. 15485350 10.1146/annurev.genom.4.070802.110305

[26] Holmes MV, Shah SH, Angelakopoulou A, Khan T, Swerdlow D, Kuchenbaecker K, et al Complex disease genetics: present and future translational applications. Genome Med. 2009;1: 104 10.1186/gm104 19891794 PMC2808739

[27] Zhao Y, Lee WN, Xiao GG. Quantitative proteomics and biomarker discovery in human cancer. Expert Rev Proteomics. 2009;6: 115–118. 10.1586/epr.09.8 19385938 PMC2835852

[28] Gobezie R, Millett PJ, Sarracino DS, Evans C, Thornhill TS. Proteomics: applications to the study of rheumatoid arthritis and osteoarthritis. J Am Acad Orthop Surg. 2006;14: 325–332. 16757672 10.5435/00124635-200606000-00002

[29] Ali M, Manolios N. Proteomics in rheumatology: a new direction for old diseases. Semin Arthritis Rheum. 2005;35: 67–76. 16194693 10.1016/j.semarthrit.2005.07.002

[30] Ritter SY, Subbaiah R, Bebek G, Crish J, Scanzello CR, Krastins B, et al Proteomic analysis of synovial fluid from the osteoarthritic knee: comparison with transcriptome analyses of joint tissues. Arthritis Rheum. 2013;65: 981–992. 10.1002/art.37823 23400684 PMC3618606

[31] Williams A, Smith JR, Allaway D, Harris P, Liddell S, Mobasheri A. Applications of proteomics in cartilage biology and osteoarthritis research. Front Biosci. 2011;16: 2622–2644. 21622199 10.2741/3876

[32] Riley G. The pathogenesis of tendinopathy. A molecular perspective. Rheumatology (Oxford). 2004;43: 131–142. 12867575 10.1093/rheumatology/keg448

[33] de Mos M, van El B, degroot J, Jahr H, van Schie HTM, van Arkel ER, et al Achilles tendinosis: Changes in biochemical composition and collagen turnover rate. Am J Sports Med. 2007;35: 1549–1556. 17478653 10.1177/0363546507301885

[34] Kmet LM, Lee RC, Cook LS, Research AHFfM (2004) Standard Quality Assessment Criteria for Evaluating Primary Research Papers from a Variety of Fields. Institute of Health Economics: Alberta Heritage Foundation for Medical Research. Available: http://www.ihe.ca/documents/HTA-FRI13.pdf. Accessed 21 November 2014.

[35] Egger M, Smith GD, Schneider M, Minder C. Bias in meta-analysis detected by a simple, graphical test. Br Med J. 1997;315: 629–634. 9310563 10.1136/bmj.315.7109.629 PMC2127453

[36] Bank RA, TeKoppele JM, Oostingh G, Hazleman BL, Riley GP. Lysylhydroxylation and non-reducible crosslinking of human supraspinatus tendon collagen: changes with age and in chronic rotator cuff tendinitis. Ann Rheum Dis. 1999;58: 35–41. 10343538 10.1136/ard.58.1.35 PMC1752756

[37] Benson RT, McDonnell SM, Rees JL, Athanasou NA, Carr AJ. The morphological and immunocytochemical features of impingement syndrome and partial-thickness rotator-cuff tear in relation to outcome after subacromial decompression. J Bone Joint Surg Br. 2009;91B: 119–123. 10.1302/0301-620X.91B1.21058 19092016

[38] Chaudhury S, Dicko C, Burgess M, Vollrath F, Carr AJ. Fourier transform infrared spectroscopic analysis of normal and torn rotator-cuff tendons. J Bone Joint Surg Br. 2011;93: 370–377. 10.1302/0301-620X.93B3.25470 21357960

[39] Hamada K, Tomonaga A, Gotoh M, Yamakawa H, Fukuda H. Intrinsic healing capacity and tearing process of torn supraspinatus tendons: In situ hybridization study of alpha 1(I) procollagen mRNA. J Orthop Res. 1997;15: 24–32. 9066523 10.1002/jor.1100150105

[40] Joseph M, Maresh CM, McCarthy MB, Kraemer WJ, Ledgard F, Arciero CL, et al Histological and Molecular Analysis of the Biceps Tendon Long Head Post-Tenotomy. J Orthop Res. 2009;27: 1379–1385. 10.1002/jor.20868 19340876

[41] Lakemeier S, Schwuchow SA, Peterlein CD, Foelsch C, Fuchs-Winkelmann S, Archontidou-Aprin E, et al Expression of matrix metalloproteinases 1, 3, and 9 in degenerated long head biceps tendon in the presence of rotator cuff tears: an immunohistological study. BMC Musculoskelet Disord. 2010;11: 271 10.1186/1471-2474-11-271 21108787 PMC2998463

[42] Lakemeier S, Braun J, Efe T, Foelsch C, Archontidou-Aprin E, Fuchs-Winkelmann S, et al Expression of matrix metalloproteinases 1, 3, and 9 in differing extents of tendon retraction in the torn rotator cuff. Knee Surg Sports Traumatol Arthrosc. 2011;19: 1760–1765. 10.1007/s00167-010-1367-y 21222105

[43] Lakemeier S, Reichelt JJ, Patzer T, Fuchs-Winkelmann S, Paletta JR, Schofer MD. The association between retraction of the torn rotator cuff and increasing expression of hypoxia inducible factor 1 and vascular endothelial growth factor expression: An immunohistological study. BMC Musculoskelet Disord. 2010;11: 230 10.1186/1471-2474-11-230 20932296 PMC2958987

[44] Lakemeier S, Reichelt JJ, Timmesfeld N, Fuchs-Winkelmann S, Paletta JR, Schofer MD. The relevance of long head biceps degeneration in the presence of rotator cuff tears. BMC Musculoskelet Disord. 2010;11: 191 10.1186/1471-2474-11-191 20799939 PMC2936349

[45] Lo IKY, Marchuk LL, Hollinshead R, Hart DA, Frank CB. Matrix metalloproteinase and tissue inhibitor of matrix metalloproteinase mRNA levels are specifically altered in torn rotator cuff tendons. Am J Sports Med. 2004;32: 1223–1229. 15262646 10.1177/0363546503262200

[46] Lo IKY, Boorman R, Marchuk L, Hollinshead R, Hart DA, Frank CB. Matrix molecule mRNA levels in the bursa and rotator cuff of patients with full-thickness rotator cuff tears. Arthroscopy. 2005;21: 645–651. 15944617 10.1016/j.arthro.2005.03.008

[47] Lundgreen K, Lian OB, Engebretsen L, Scott A. Tenocyte apoptosis in the torn rotator cuff: a primary or secondary pathological event? Br J Sports Med. 2011;45: 1035–1039. 10.1136/bjsm.2010.083188 21482545 PMC3951987

[48] Millar NL, Wei AQ, Molloy TJ, Bonar F, Murrell GA. Cytokines and apoptosis in supraspinatus tendinopathy. J Bone Joint Surg Br. 2009;91: 417–424. 10.1302/0301-620X.91B3.21652 19258623

[49] Oliva F, Zocchi L, Codispoti A, Candi E, Celi M, Melino G, et al Transglutaminases expression in human supraspinatus tendon ruptures and in mouse tendons. Biochem Biophys Res Commun. 2009;379: 887–891. 10.1016/j.bbrc.2008.12.188 19146825

[50] Riley GP, Harrall H, Constant CR, Chard MD, Cawston TE, Hazleman BL. Tendon degeneration and chronic shoulder pain: Changes in the collagen composition of the human rotator cuff tendons in rotator cuff tendinitis. Ann Rheum Dis. 1994;53: 359–366. 8037494 10.1136/ard.53.6.359 PMC1005350

[51] Riley GP, Curry V, DeGroot J, van El B, Verzijl N, Hazleman BL, et al Matrix metalloproteinase activities and their relationship with collagen remodelling in tendon pathology. Matrix Biol. 2002;21: 185–195. 11852234 10.1016/s0945-053x(01)00196-2

[52] Shindle MK, Chen CCT, Robertson C, DiTullio AE, Paulus MC, Clinton CM, et al Full-thickness supraspinatus tears are associated with more synovial inflammation and tissue degeneration than partial-thickness tears. J Shoulder Elbow Surg. 2011;20: 917–927. 10.1016/j.jse.2011.02.015 21612944 PMC3156316

[53] Shirachi I, Gotoh M, Mitsui Y, Yamada T, Nakama K, Kojima K, et al Collagen production at the edge of ruptured rotator cuff tendon is correlated with postoperative cuff integrity. Arthroscopy. 2011;27: 1173–1179. 10.1016/j.arthro.2011.03.078 21752571

[54] Singaraju VM, Kang RW, Yanke AB, McNickle AG, Lewis PB, Wang VM, et al Biceps tendinitis in chronic rotator cuff tears: a histologic perspective. J Shoulder Elbow Surg. 2008;17: 898–904. 10.1016/j.jse.2008.05.044 18786837

[55] Tillander B, Franzen L, Norlin R. Fibronectin, MMP-1 and histologic changes in rotator cuff disease. J Orthop Res. 2002;20: 1358–1364. 12472253 10.1016/S0736-0266(02)00057-8

[56] Tomonaga A, Hamada K, Gotoh M, Yamakawa H, Kobayashi K, Fukuda H. Expression of procollagen alpha 1 type III mRNA in rotator cuff tears. Tokai J Exp Clin Med. 2000;25: 125–134. 11368210

[57] Wang MX, Wei AQ, Yuan J, Clippe A, Bernard A, Knoops B, et al Antioxidant enzyme peroxiredoxin 5 is upregulated in degenerative human tendon. Biochem Biophys Res Commun. 2001;284: 667–673. 11396953 10.1006/bbrc.2001.4991

[58] Qi J, Dmochowski JM, Banes AN, Tsuzaki M, Bynum D, Patterson M, et al Differential expression and cellular localization of novel isoforms of the tendon biomarker tenomodulin. J Appl Physiol. 2012;113: 861–871. 10.1152/japplphysiol.00198.2012 22700804

[59] Alfredson H, Lorentzon M, Backman S, Backman A, Lerner UH. cDNA-arrays and real-time quantitative PCR techniques in the investigation of chronic achilles tendinosis. J Orthop Res. 2003;21: 970–975. 14554207 10.1016/S0736-0266(03)00107-4

[60] Bjorklund E, Forsgren S, Alfredson H, Fowler CJ. Increased expression of cannabinoid CB(1) receptors in Achilles tendinosis. PLoS One. 2011;6: e24731 10.1371/journal.pone.0024731 21931835 PMC3169627

[61] Corps AN, Robinson AHN, Movin T, Costa ML, Ireland DC, Hazleman BL, et al Versican splice variant messenger RNA expression in normal human Achilles tendon and tendinopathies. Rheumatology (Oxford). 2004;43: 969–972. 15138331 10.1093/rheumatology/keh222

[62] Corps AN, Robinson AHN, Movin T, Costa ML, Hazleman BL, Riley GP. Increased expression of aggrecan and biglycan mRNA in Achilles tendinopathy. Rheumatology (Oxford). 2006;45: 291–294. 16219640 10.1093/rheumatology/kei152

[63] Corps AN, Jones GC, Harrall RL, Curry VA, Hazleman BL, Riley GP. The regulation of aggrecanase ADAMTS-4 expression in human Achilles tendon and tendon-derived cells. Matrix Biol. 2008;27: 393–401. 10.1016/j.matbio.2008.02.002 18387286 PMC2443387

[64] Eriksen HA, Pajala A, Leppilahti J, Risteli J. Increased content of type III collagen at the rupture site of human Achilles tendon. J Orthop Res. 2002;20: 1352–1357. 12472252 10.1016/S0736-0266(02)00064-5

[65] Fenwick SA, Curry V, Harrall RL, Hazleman BL, Hackney R, Riley GP. Expression of transforming growth factor-beta isoforms and their receptors in chronic tendinosis. J Anat. 2001;199: 231–240. 11554502 10.1046/j.1469-7580.2001.19930231.x PMC1468327

[66] Ireland D, Harrall R, Curry V, Holloway G, Hackney R, Hazleman B, et al Multiple changes in gene expression in chronic human Achilles tendinopathy. Matrix Biol. 2001;20: 159–169. 11420148 10.1016/s0945-053x(01)00128-7

[67] Jones GC, Corps AN, Pennington CJ, Clark IM, Edwards DR, Bradley MM, et al Expression profiling of metalloproteinases and tissue inhibitors of metalloproteinases in normal and degenerate human Achilles tendon. Arthritis Rheum. 2006;54: 832–842. 16508964 10.1002/art.21672

[68] Karousou E, Ronga M, Vigetti D, Passi A, Maffulli N. Collagens, proteoglycans, MMP-2, MMP-9 and TIMPs in human achilles tendon rupture. Clin Orthop Relat Res. 2008;466: 1577–1582. 10.1007/s11999-008-0255-y 18425559 PMC2505242

[69] Pajala A, Melkko J, Leppilahti J, Ohtonen P, Soini Y, Risteli J. Tenascin-C and type I and III collagen expression in total Achilles tendon rupture. An immunohistochemical study. Histol Histopathol. 2009;24: 1207–1211. 19688689 10.14670/HH-24.1207

[70] Pingel J, Fredberg U, Qvortrup K, Larsen JO, Schjerling P, Heinemeier K, et al Local biochemical and morphological differences in human Achilles tendinopathy: a case control study. BMC Musculoskelet Disord. 2012;13: 53 10.1186/1471-2474-13-53 22480275 PMC3341204

[71] Pufe T, Petersen W, Tillmann B, Mentlein R. The angiogenic peptide vascular endothelial growth factor is expressed in foetal and ruptured tendons. Virchows Arch. 2001;439: 579–585. 11710646 10.1007/s004280100422

[72] Bridgeman JT, Zhang Y, Donahue H, Wade AM, Juliano PJ. Estrogen Receptor Expression in Posterior Tibial Tendon Dysfunction: A Pilot Study. Foot Ankle Int. 2010;31: 1081–1084. 10.3113/FAI.2010.1081 21189209

[73] Corps AN, Robinson AH, Harrall RL, Avery NC, Curry VA, Hazleman BL, et al Changes in matrix protein biochemistry and the expression of mRNA encoding matrix proteins and metalloproteinases in posterior tibialis tendinopathy. Ann Rheum Dis. 2012;71: 746–752. 10.1136/annrheumdis-2011-200391 22241901 PMC3329235

[74] Goncalves-Neto J, Witzel SS, Teodoro WR, Carvalho-Junior AE, Fernandes TD, Yoshinari HH. Changes in collagen matrix composition in human posterior tibial tendon dysfunction. Joint Bone Spine. 2002;69: 189–194. 12027311 10.1016/s1297-319x(02)00369-x

[75] Fu SC, Chan BP, Wang W, Pau HM, Chan KM, Rolf CG. Increased expression of matrix metalloproteinase 1 (MMP1) in 11 patients with patellar tendinosis. Acta Orthop Scand. 2002;73: 658–662. 12553513 10.1080/000164702321039624

[76] Fu SC, Wang W, Pau HM, Wong YP, Chan KM, Rolf CG. Increased expression of transforming growth factor-beta 1 in patellar tendinosis. Clin Orthop Relat Res. 2002: 174–183. 12072760 10.1097/00003086-200207000-00022

[77] Fu SC, Chan KM, Rolf CG. Increased deposition of sulfated glycosaminoglycans in human patellar tendinopathy. Clin J Sport Med. 2007;17: 129–134. 17414481 10.1097/JSM.0b013e318037998f

[78] Parkinson J, Samiric T, Ilic MZ, Cook J, Feller JA, Handley CJ. Change in Proteoglycan Metabolism Is a Characteristic of Human Patellar Tendinopathy. Arthritis Rheum. 2010;62: 3028–3035. 10.1002/art.27587 20533294

[79] Samiric T, Parkinson J, Ilic MZ, Cook J, Feller JA, Handley CJ. Changes in the composition of the extracellular matrix in patellar tendinopathy. Matrix Biol. 2009;28: 230–236. 10.1016/j.matbio.2009.04.001 19371780

[80] Schizas N, Lian O, Frihagen F, Engebretsen L, Bahr R, Ackermann PW. Coexistence of up-regulated NMDA receptor 1 and glutamate on nerves, vessels and transformed tenocytes in tendinopathy. Scand J Med Sci Sports. 2010;20: 208–215. 10.1111/j.1600-0838.2009.00913.x 19422642

[81] Schizas N, Weiss R, Lian O, Frihagen F, Bahr R, Ackermann PW. Glutamate receptors in tendinopathic patients. J Orthop Res. 2012;30: 1447–1452. 10.1002/jor.22094 22354721

[82] Scott A, Lian O, Bahr R, Hart DA, Duronio V. VEGF expression in patellar tendinopathy: a preliminary study. Clin Orthop Relat Res. 2008;466: 1598–1604. 10.1007/s11999-008-0272-x 18459027 PMC2505256

[83] Scott A, Lian O, Roberts CR, Cook JL, Handley CJ, Bahr R, et al Increased versican content is associated with tendinosis pathology in the patellar tendon of athletes with jumper's knee. Scand J Med Sci Sports. 2008;18: 427–435. 18067512 10.1111/j.1600-0838.2007.00735.x PMC3951986

[84] Scott A, Alfredson H, Forsgren S. VGluT2 expression in painful Achilles and patellar tendinosis: Evidence of local glutamate release by tenocytes. J Orthop Res. 2008;26: 685–692. 18050306 10.1002/jor.20536 PMC3951483

[85] Legerlotz K, Jones ER, Screen HRC, Riley GP. Increased expression of IL-6 family members in tendon pathology. Rheumatology (Oxford). 2012;51: 1161–1165. 10.1093/rheumatology/kes002 22337942 PMC3380247

[86] Jelinsky SA, Rodeo SA, Li J, Gulotta LV, Archambault JM, Seeherman HJ. Regulation of gene expression in human tendinopathy. BMC Musculoskelet Disord. 2011;12: 86 10.1186/1471-2474-12-86 21539748 PMC3095578

[87] Ohlendieck K. Proteomic profiling of skeletal muscle plasticity. Muscles Ligaments Tendons J. 2011;1: 119–126. 23738259 PMC3666486

[88] Sodersten F, Ekman S, Schmitz M, Paulsson M, Zaucke F. Thrombospondin-4 and cartilage oligomeric matrix protein form heterooligomers in equine tendon. Connect Tissue Res. 2006;47: 85–91. 16754514 10.1080/03008200600584124

[89] Xia W, Wang Y, Appleyard RC, Smythe GA, Murrell GA. Spontaneous recovery of injured Achilles tendon in inducible nitric oxide synthase gene knockout mice. Inflamm Res. 2006;55: 40–45. 16429255 10.1007/s00011-005-0006-4

[90] Yoon JH, Brooks RL Jr., Zhao JZ, Isaacs D, Halper J. The effects of enrofloxacin on decorin and glycosaminoglycans in avian tendon cell cultures. Arch Toxicol. 2004;78: 599–608. 15148565 10.1007/s00204-004-0574-z

[91] Harris RD, Nindl G, Balcavage WX, Weiner W, Johnson MT. Use of proteomics methodology to evaluate inflammatory protein expression in tendinitis. Biomed Sci Instrum. 2003;39: 493–499. 12724941

[92] Hakimi O, Ternette N, Murphy R, Kessler B, Carr A. The proteome of young and healthy human hamstring. Osteoarthritis Cartilage. 2012;20: S262–S263.

[93] MacIej ML, Hyde GD, Boot-Handford RP, Wallis GA, Kadler KE. Type II collagen expression in tendon: A new look at tendon development and repair. Eur Cell Mater. 2011;22: 64.

[94] Jielile J, Jialili A, Sabirhazi G, Shawutali N, Redati D, Chen J, et al Proteomic analysis of differential protein expression of achilles tendon in a rabbit model by two-dimensional polyacrylamide gel electrophoresis at 21 days postoperation. Appl Biochem Biotechnol. 2011;165: 1092–1106. 10.1007/s12010-011-9327-7 21800109

[95] Todhunter RJ, Wootton JAM, Lust G, Minor RR. Structure of Equine Type-I and Type-Ii Collagens. Am J Vet Res. 1994;55: 425–431. 8192271

[96] Yoon JH, Brooks RL, Khan A, Pan H, Bryan J, Zhang J, et al The effect of enrofloxacin on cell proliferation and proteoglycans in horse tendon cells. Cell Biol Toxicol. 2004;20: 41–54. 15119847 10.1023/b:cbto.0000021154.01035.f9

[97] Kim B, Yoon JH, Zhang JA, Mueller POE, Halper J. Glycan profiling of a defect in decorin glycosylation in equine systemic proteoglycan accumulation, a potential model of progeroid form of Ehlers-Danlos syndrome. Arch Biochem Biophys. 2010;501: 221–231. 10.1016/j.abb.2010.06.017 20599673

[98] Johnson MT. Proteomics of tendinopathy. Front Biosci. 2009;14: 1505–1515. 19273143 10.2741/3321

[99] Jiang Y, Liu H, Li H, Wang F, Cheng K, Zhou G, et al A proteomic analysis of engineered tendon formation under dynamic mechanical loading in vitro. Biomaterials. 2011;32: 4085–4095. 10.1016/j.biomaterials.2011.02.033 21402406

[100] Miller BF, Olesen JL, Hansen M, Dossing S, Crameri RM, Welling RJ, et al Coordinated collagen and muscle protein synthesis in human patella tendon and quadriceps muscle after exercise. J Physiol. 2005;567: 1021–1033. 16002437 10.1113/jphysiol.2005.093690 PMC1474228

[101] Rolf CG, Fu BS, Pau A, Wang W, Chan B. Increased cell proliferation and associated expression of PDGFRbeta causing hypercellularity in patellar tendinosis. Rheumatology (Oxford). 2001;40: 256–261. 11285371 10.1093/rheumatology/40.3.256

[102] Sato N, Taniguchi T, Goda Y, Kosaka H, Higashino K, Sakai T, et al Solubilization and analysis of insoluble extracellular matrix: Proteomic analysis of human tendon and ligament. Connect Tissue Res. 2012;53 (1): 59–60. 10.1021/acs.jproteome.6b00806 27748110

[103] Riley GP, Harrall RL, Constant CR, Chard MD, Cawston TE, Hazleman BL. Tendon Degeneration and Chronic Shoulder Pain—Changes in the Collagen Composition of the Human Rotator Cuff Tendons in Rotator Cuff Tendinitis. Ann Rheum Dis. 1994;53: 359–366. 8037494 10.1136/ard.53.6.359 PMC1005350

[104] Benson RT, McDonnell SM, Knowles HJ, Rees JL, Carr AJ, Hulley PA. Tendinopathy and tears of the rotator cuff are associated with hypoxia and apoptosis. J Bone Joint Surg Br. 2010;92B: 448–453. 10.1302/0301-620X.92B3.23074 20190320 PMC2843163

[105] Dean BJ, Franklin SL, Carr AJ. A systematic review of the histological and molecular changes in rotator cuff disease. Bone Joint Res. 2012;1: 158–166. 10.1302/2046-3758.17.2000115 23610686 PMC3626275

[106] Gotoh M, Hamada K, Yamakawa H, Yanagisawa K, Nakamura M, Yamazaki H, et al Interleukin-1-induced subacromial synovitis and shoulder pain in rotator cuff diseases. Rheumatology (Oxford). 2001;40: 995–1001. 11561109 10.1093/rheumatology/40.9.995

[107] Voloshin I, Gelinas J, Maloney MD, O'Keefe RJ, Bigliani LU, Blaine TA. Proinflammatory cytokines and metalloproteases are expressed in the subacromial bursa in patients with rotator cuff disease. Arthroscopy. 2005;21: 1076 e1071–1076 e1079 16171632 10.1016/j.arthro.2005.05.017

[108] Berenson MC, Blevins FT, Plaas AHK, Vogel KG. Proteoglycans of human rotator cuff tendons. J Orthop Res. 1996;14: 518–525. 8764859 10.1002/jor.1100140404

[109] Benjamin M, Kaiser E, Milz S. Structure-function relationships in tendons: a review. J Anat. 2008;212: 211–228. 10.1111/j.1469-7580.2008.00864.x 18304204 PMC2408985

[110] Kjaer M. Role of extracellular matrix in adaptation of tendon and skeletal muscle to mechanical loading. Physiol Rev. 2004;84: 649–698. 15044685 10.1152/physrev.00031.2003

[111] Matuszewski PE, Chen YL, Szczesny SE, Lake SP, Elliott DM, Soslowsky LJ, et al Regional Variation in Human Supraspinatus Tendon Proteoglycans: Decorin, Biglycan, and Aggrecan. Connect Tissue Res. 2012;53: 343–348. 10.3109/03008207.2012.654866 22329809 PMC3437000

[112] Yamamoto A, Takagishi K, Osawa T, Yanagawa T, Nakajima D, Shitara H, et al Prevalence and risk factors of a rotator cuff tear in the general population. J Shoulder Elbow Surg. 2010;19: 116–120. 10.1016/j.jse.2009.04.006 19540777

[113] Yamaguchi K, Ditsios K, Middleton WD, Hildebolt CF, Galatz LM, Teefey SA. The demographic and morphological features of rotator cuff disease. A comparison of asymptomatic and symptomatic shoulders. J Bone Joint Surg Am. 2006;88: 1699–1704. 16882890 10.2106/JBJS.E.00835

[114] Hijioka A, Suzuki K, Nakamura T, Hojo T. Degenerative change and rotator cuff tears. An anatomical study in 160 shoulders of 80 cadavers. Arch Orthop Trauma Surg. 1993;112: 61–64. 8457412 10.1007/BF00420255

[115] Lehman C, Cuomo F, Kummer FJ, Zuckerman JD. The incidence of full thickness rotator cuff tears in a large cadaveric population. Bull Hosp Jt Dis. 1995;54: 30–31. 8541777

[116] Riley GP, Harrall RL, Constant CR, Chard MD, Cawston TE, Hazleman BL. Rotator cuff tendinitis: Changes in the collagen composition of the human rotator cuff tendons with age and in disease. Int J Exp Pathol. 1994;75: A29–A30. 10.1136/ard.53.6.359 PMC1005350 8037494

[117] Junge K, Klinge U, Rosch R, Mertens PR, Kirch J, Klosterhalfen B, et al Decreased collagen type I/III ratio in patients with recurring hernia after implantation of alloplastic prostheses. Langenbecks Arch Surg. 2004;389: 17–22. 14576942 10.1007/s00423-003-0429-8

[118] Sharma P, Maffulli N. Tendon injury and tendinopathy: healing and repair. J Bone Joint Surg Am. 2005;87: 187–202. 15634833 10.2106/JBJS.D.01850

[119] Couppe C, Hansen P, Kongsgaard M, Kovanen V, Suetta C, Aagaard P, et al Mechanical properties and collagen cross-linking of the patellar tendon in old and young men. J Appl Physiol. 2009;107: 880–886. 10.1152/japplphysiol.00291.2009 19556458

[120] Bank RA, Bayliss MT, Lafeber FP, Maroudas A, Tekoppele JM. Ageing and zonal variation in post-translational modification of collagen in normal human articular cartilage. The age-related increase in non-enzymatic glycation affects biomechanical properties of cartilage. Biochem J. 1998;330 (Pt 1): 345–351. 9461529 10.1042/bj3300345 PMC1219146

[121] Garofalo R, Cesari E, Vinci E, Castagna A. Role of metalloproteinases in rotator cuff tear. Sports Med Arthrosc. 2011;19: 207–212. 10.1097/JSA.0b013e318227b07b 21822103

[122] Rodriguez D, Morrison CJ, Overall CM. Matrix metalloproteinases: what do they not do? New substrates and biological roles identified by murine models and proteomics. Biochim Biophys Acta. 2010;1803: 39–54. 10.1016/j.bbamcr.2009.09.015 19800373

[123] Magra M, Maffulli N. Matrix metalloproteases: a role in overuse tendinopathies. Br J Sports Med. 2005;39: 789–791. 16244185 10.1136/bjsm.2005.017855 PMC1725078

[124] Pasternak B, Aspenberg P. Metalloproteinases and their inhibitors-diagnostic and therapeutic opportunities in orthopedics. Acta Orthop. 2009;80: 693–703. 10.3109/17453670903448257 19968600 PMC2823312

[125] Visse R, Nagase H. Matrix metalloproteinases and tissue inhibitors of metalloproteinases: structure, function, and biochemistry. Circ Res. 2003;92: 827–839. 12730128 10.1161/01.RES.0000070112.80711.3D

[126] Riley GP, Harrall RL, Constant CR, Chard MD, Cawston TE, Hazleman BL. Glycosaminoglycans of human rotator cuff tendons: changes with age and in chronic rotator cuff tendinitis. Ann Rheum Dis. 1994;53: 367–376. 8037495 10.1136/ard.53.6.367 PMC1005351

[127] Benjamin M, Ralphs JR. Fibrocartilage in tendons and ligaments—an adaptation to compressive load. J Anat. 1998;193 (Pt 4): 481–494. 10029181 10.1046/j.1469-7580.1998.19340481.x PMC1467873

[128] Robbins JR, Evanko SP, Vogel KG. Mechanical loading and TGF-beta regulate proteoglycan synthesis in tendon. Arch Biochem Biophys. 1997;342: 203–211. 9186480 10.1006/abbi.1997.0102

[129] Martin JA, Mehr D, Pardubsky PD, Buckwalter JA. The role of tenascin-C in adaptation of tendons to compressive loading. Biorheology. 2003;40: 321–329. 12454422

[130] Mehr D, Pardubsky PD, Martin JA, Buckwalter JA. Tenascin-C in tendon regions subjected to compression. J Orthop Res. 2000;18: 537–545. 11052489 10.1002/jor.1100180405

[131] Jarvinen TAH, Jozsa L, Kannus P, Jarvinen TLN, Kvist M, Hurme T, et al Mechanical loading regulates tenascin-C expression in the osteotendinous junction. J Cell Sci. 1999;112: 3157–3166. 10462531 10.1242/jcs.112.18.3157

[132] Jarvinen TAH, Kannus P, Jarvinen TLN, Jozsa L, Kalimo H, Jarvinen M. Tenascin-C in the pathobiology and healing process of musculoskeletal tissue injury. Scand J Med Sci Sports. 2000;10: 376–382. 11085568 10.1034/j.1600-0838.2000.010006376.x

[133] Jarvinen TA, Jozsa L, Kannus P, Jarvinen TL, Hurme T, Kvist M, et al Mechanical loading regulates the expression of tenascin-C in the myotendinous junction and tendon but does not induce de novo synthesis in the skeletal muscle. J Cell Sci. 2003;116: 857–866. 12571283 10.1242/jcs.00303

[134] Chiquet-Ehrismann R, Tannheimer M, Koch M, Brunner A, Spring J, Martin D, et al Tenascin-C expression by fibroblasts is elevated in stressed collagen gels. J Cell Biol. 1994;127: 2093–2101. 7528751 10.1083/jcb.127.6.2093 PMC2120287

[135] Patel L, Sun W, Glasson SS, Morris EA, Flannery CR, Chockalingam PS. Tenascin-C induces inflammatory mediators and matrix degradation in osteoarthritic cartilage. BMC Musculoskelet Disord. 2011;12: 164 10.1186/1471-2474-12-164 21762512 PMC3146914

[136] Cook JL, Purdam CR. Is tendon pathology a continuum? A pathology model to explain the clinical presentation of load-induced tendinopathy. Br J Sports Med. 2009;43: 409–416. 10.1136/bjsm.2008.051193 18812414

[137] Battery L, Maffulli N. Inflammation in overuse tendon injuries. Sports Med Arthrosc. 2011;19: 213–217. 10.1097/JSA.0b013e31820e6a92 21822104

[138] Backman LJ, Andersson G, Wennstig G, Forsgren S, Danielson P. Endogenous substance P production in the Achilles tendon increases with loading in an in vivo model of tendinopathy—peptidergic elevation preceding tendinosis-like tissue changes. J Musculoskelet Neuronal Interact. 2011;11: 133–140. 21625050

[139] Bagge J, Gaida JE, Danielson P, Alfredson H, Forsgren S. Physical activity level in Achilles tendinosis is associated with blood levels of pain-related factors: a pilot study. Scand J Med Sci Sports. 2011;21: E430–E438. 10.1111/j.1600-0838.2011.01358.x 21819445

[140] Legerlotz K, Jones GC, Screen HR, Riley GP. Cyclic loading of tendon fascicles using a novel fatigue loading system increases interleukin-6 expression by tenocytes. Scand J Med Sci Sports. 2011;23: 31–37. 10.1111/j.1600-0838.2011.01410.x 22092479 PMC3558793

[141] Maeda T, Sakabe T, Sunaga A, Sakai K, Rivera AL, Keene DR, et al Conversion of mechanical force into TGF-beta-mediated biochemical signals. Curr Biol. 2011;21: 933–941. 10.1016/j.cub.2011.04.007 21600772 PMC3118584

[142] Alfredson H. Strategies in treatment of tendon overuse injury. The chronic painful tendon. European Journal of Sport Science. 2006;6: 81–85.

[143] Backman LJ, Andersson G, Wennstig G, Forsgren S, Danielson P. Endogenous substance P production in the Achilles tendon increases with loading in an in vivo model of tendinopathy-peptidergic elevation preceding tendinosis-like tissue changes. J Musculoskelet Neuronal Interact. 2011;11: 133–140. 21625050

[144] Schubert TE, Weidler C, Lerch K, Hofstadter F, Straub RH. Achilles tendinosis is associated with sprouting of substance P positive nerve fibres. Ann Rheum Dis. 2005;64: 1083–1086. 15958764 10.1136/ard.2004.029876 PMC1755550

[145] Danielson P, Alfredson H, Forsgren S. Distribution of general (PGP 9.5) and sensory (substance P/CGRP) innervations in the human patellar tendon. Knee Surg Sports Traumatol Arthrosc. 2006;14: 125–132. 15983834 10.1007/s00167-005-0636-7

[146] Tsuzaki M, Guyton G, Garrett W, Archambault JM, Herzog W, Almekinders L, et al IL-1 beta induces COX2, MMP-1, -3 and -13, ADAMTS-4, IL-1 beta and IL-6 in human tendon cells. J Orthop Res. 2003;21: 256–264. 12568957 10.1016/S0736-0266(02)00141-9

[147] Lin TW, Cardenas L, Glaser DL, Soslowsky LJ. Tendon healing in interleukin-4 and interleukin-6 knockout mice. J Biomech. 2006;39: 61–69. 16271588 10.1016/j.jbiomech.2004.11.009

[148] Evans CH. Cytokines and the role they play in the healing of ligaments and tendons. Sports Med. 1999;28: 71–76. 10492026 10.2165/00007256-199928020-00001

[149] Chen CH, Cao Y, Wu YF, Bais AJ, Gao JS, Tang JB. Tendon healing in vivo: gene expression and production of multiple growth factors in early tendon healing period. J Hand Surg Am. 2008;33: 1834–1842. 10.1016/j.jhsa.2008.07.003 19084187

[150] Birch HL. Tendon matrix composition and turnover in relation to functional requirements. Int J Exp Pathol. 2007;88: 241–248. 17696905 10.1111/j.1365-2613.2007.00552.x PMC2517317

[151] Neer CS 2nd. Anterior acromioplasty for the chronic impingement syndrome in the shoulder. 1972. J Bone Joint Surg Am. 2005;87: 1399 15930554 10.2106/JBJS.8706.cl

[152] Hammer E, Goritzka M, Ameling S, Darm K, Steil L, Klingel K, et al Characterization of the human myocardial proteome in inflammatory dilated cardiomyopathy by label-free quantitative shotgun proteomics of heart biopsies. J Proteome Res. 2011;10: 2161–2171. 10.1021/pr1008042 21417265

[153] Aebersold R, Mann M. Mass spectrometry-based proteomics. Nature. 2003;422: 198–207. 12634793 10.1038/nature01511

[154] Ong SE, Mann M. Mass spectrometry-based proteomics turns quantitative. Nat Chem Biol. 2005;1: 252–262. 16408053 10.1038/nchembio736

[155] Bantscheff M, Lemeer S, Savitski MM, Kuster B. Quantitative mass spectrometry in proteomics: critical review update from 2007 to the present. Anal Bioanal Chem. 2012;404: 939–965. 10.1007/s00216-012-6203-4 22772140

[156] Bantscheff M, Schirle M, Sweetman G, Rick J, Kuster B. Quantitative mass spectrometry in proteomics: a critical review. Anal Bioanal Chem. 2007;389: 1017–1031. 17668192 10.1007/s00216-007-1486-6

