# Supplementary figures and images for: Proteomics Perspectives in Rotator Cuff Research: A Systematic Review of Gene Expression and Protein Composition in Human Tendinopathy
Source: PLoS One. 2015 Apr 16;10(4):e0119974. doi: 10.1371/journal.pone.0119974 (PMC4400011; doi:10.1371/journal.pone.0119974)

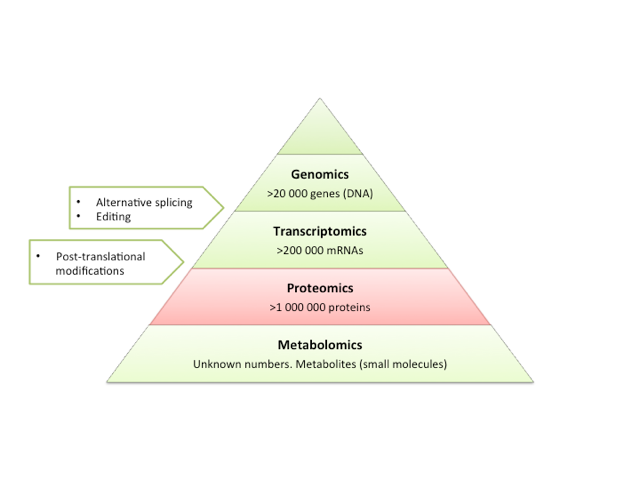

Supplement: S1 Fig — The complexity and size of each compartment increase with each step down the pyramid. Adapted from Holmes et al[26]. (TIFF) [file pone.0119974.s001.tiff]
