# Supplementary material for: Proteomics Perspectives in Rotator Cuff Research: A Systematic Review of Gene Expression and Protein Composition in Human Tendinopathy
Source: PLoS One. 2015 Apr 16;10(4):e0119974. doi: 10.1371/journal.pone.0119974 (PMC4400011; doi:10.1371/journal.pone.0119974)
Supplement: S1 Table — None of the studies that quantified proteins used proteomics technologies. Two authors in same row indicate that the same patient and control populations were used in the two studies; () = non-significant trend. (DOCX) [file pone.0119974.s002.docx]

**S1 Table. Gene expression and protein composition in patellar tendinopathy.** None of the studies that quantified proteins used proteomics technologies. Two authors in same row indicate that the same patient and control populations were used in the two studies; ()=non-significant trend.

|  |  | |  |  | | |  | |
| --- | --- | --- | --- | --- | --- | --- | --- | --- |
|  | **Sample setting, anatomical site of sample, diagnosis, number of patients (n), mean age (range)** | |  | **Direction of change of target tendon components** | | |  | |
|  |  | |  |  | | |  | |
|  |  |  |  |  |  |  |  |  |
| **First author, year** | **Patient samples** | **Control samples** | **Method** | **Up** | **Down** | **No difference** | **Comment** | **Quality** |
|  |  |  |  |  |  |  |  | **Score** |
| **Fu, 2002a**[75] | Peroperative, pt | Peroperative, pt, normal from | Proteins | MMP-1, | TIMP-1 | Decorin, | Quantification of percentage | 85 |
| **Fu, 2002b**[76] | tendinopathy, n=11, 31 | patients undergoing ACL |  | procollagen I, |  | biglycan | of immuno-positive cells. |  |
| **Fu, 2007**[77] | (15-40) | reconstruction, n=12, 31 (16-38) |  | COX-2, TGF-β1, Proteoglycans |  |  | Results from in vitro study are not included in this table |  |
| **Parkinson, 2010**[78] | Peroperative, pt, tendinopathy, n=12, n.r. (n.r.) | Peroperative, pt, normal from patients undergoing ACL reconstruction, n=9, n.r. (n.r.) | Tran-script | MMP-9, TIMP-1 | (MMP-3) | ADAMTS-1,-4,-5, MMP-1,-2,-13, TIMP-2,-3,-4 | RT-PCR normalised to GADPH. Results from explant culture are not included in this table | 75 |
| **Samiric, 2009**[79] | Peroperative, pt, tendinopathy, n=24, 27.1 (17-38) | Peroperative, pt, normal from patients undergoing ACL reconstruction, n=25, 28.0 (17-49) | Tran-scripts | (Collagen III, versican) | (Collagen I, fibromodulin) | Collagen II, decorin, biglycan, fibromodulin, versican, aggrecan | RT-PCR normalised to GADPH | 92 |
|  |  |  | Proteins | Versican, aggrecan, biglycan, fibromodulin | - | Collagen, decorin | ()=near-significant |  |
| **Schizas, 2010**[80] | Peroperative, pt, | Peroperative, pt, normal from | Proteins | NMDAR1, | - | mGluR6-7 | Semiquantitative analysis | 92 |
| **Schizas, 2012**[81] | tendinopathy, n=10, 28 (19-32) | patients with tibial shaft fractures, n=8, 32 (19-60) |  | pNMDAR1, SP, mGluR5, Glutamate |  |  | based on mean fluorescence/total area or number of signal-positive cells per objective field performed by two independent, blinded observers |  |
| **Scott, 2008a**[82] | Peroperative, pt, tendinopathy, n=22, 30.4 (22-40) | Peroperative, pt, normal from patients with tibial shaft fractures, n=10, 28.2 (21-42) | Proteins | VEGF | - | - | Quantification based on number of fields with positive staining | 85 |
| **Scott, 2008b**[83] | Peroperative, pt, tendinopathy, n=21, 30 (24-34) | Peroperative, pt, normal from patients with tibial shaft fractures, n=10, 29 (19-43) | Proteins | Versican | - | - |  | 88 |

Abbreviations: ACL=anterior cruciate ligament, ADAMTS=a disintegrin and metalloproteinase with thrombostin motifs, COX=cyclooxygenase, GADPH=glyceraldehyde 3-phosphate dehydrogenase, mGluR=metabotropic glutamate receptor, MMP=matrix metalloproteinase, NMDAR=N-methyl-D-aspartate receptor, n.r.=not reported, pNMDAR=phospho-NMDAR, pt=patellar tendon, SP=substance P, TGF-β=transforming growth factor-β, TIMP=tissue inhibitor of metalloproteinases, VEGF=vascular endothelial growth factor
