# Supplementary material for: Proteomics Perspectives in Rotator Cuff Research: A Systematic Review of Gene Expression and Protein Composition in Human Tendinopathy
Source: PLoS One. 2015 Apr 16;10(4):e0119974. doi: 10.1371/journal.pone.0119974 (PMC4400011; doi:10.1371/journal.pone.0119974)
Supplement: S2 Table — None of the studies that quantified proteins used proteomics technologies. Two authors in same row indicate that the same patient and control populations were used in the two studies; () = non-significant trend, [] = could not be detected in all samples. (DOCX) [file pone.0119974.s003.docx]

**S2 Table. Gene expression and protein composition in Achilles tendinopathy and ruptures.** None of the studies that quantified proteins used proteomics technologies. Two authors in same row indicate that the same patient and control populations were used in the two studies; ()=non-significant trend, []=could not be detected in all samples.

|  |  | |  |  | | |  | |
| --- | --- | --- | --- | --- | --- | --- | --- | --- |
|  | **Sample setting, anatomical site of sample, diagnosis, number of patients (n), mean age (range)** | |  | **Direction of change of target tendon components** | | |  | |
|  |  | |  |  | | |  | |
|  |  |  |  |  |  |  |  |  |
| **First author, year** | **Patient samples** | **Control samples** | **Method** | **Up** | **Down** | **No difference** | **Comment** | **Quality** |
|  |  |  |  |  |  |  |  | **Score** |
| **Alfredson, 2003**[59] | Peroperative, ach, chronic painful, n=5, 57.1, (n.r.) | Peroperative, ach, normal from same patient | Tran-scripts | MMP-2, FNRB, VEGF, MAPKp38 | MMP-3, decorin | IL-1-6, IL-10-15, ILR-1-7, ILR-9-12, GM-CSF, G-CSF | cDNA arrays and RT-PCR were performed; only marginal differences in MAPKp38 and decorin were detected by RT-PCR | 71 |
| **Bjorklund, 2011**[60] | Peroperative, ach, tendinopathy, n=17, 51 (28-70) | Peroperative, ach, normal, n=7, 41 (21-47) | Proteins | CB_1_R | - | - | Semiquantitative determination of CB_1_R immuno-reactivity by two independent, blinded researchers | 88 |
| **Corps, 2004**[61] | Peroperative, ach, chronic painful, n=10, 44 (32-58) | Cadaver, ach, normal, n=14, 58 (20-97) | Tran-scripts | Collagen I α1 | Versican | - | RT-PCR normalised to GADPH | 71 |
|  | Peroperative, ach, rupture, n=8, 47 (33-69) |  |  |  |  |  |  |  |
| **Corps, 2006**[62] | I: Peroperative, ach, | II: Cadaver, ach, normal, n=14, | Tran- | Aggrecan, | - | Versican, | RT-PCR normalised to 18s | 71 |
| **Corps, 2008**[63] | chronic painful, n=13, 43 (32-59) | 55 (20-97) | scripts | biglycan |  | decorin, ADAMTS-4 | rRNA |  |
|  | Ia: Peroperative, ach, rupture, n=14, 45 (25-69) | II | Proteins | - | - | ADAMTS-4 | Results from in vitro study are not included in this table |  |
|  |  |  | Tran-scripts | ADAMTS-4 | Decorin | Aggrecan, biglycan, versican |  |  |
|  |  |  | Proteins | ADAMTS-4 | - | - |  |  |
| **De Mos, 2007**[33] | Peroperative, ach, tendinopathy, n=10, 46 (36-58) | Peroperative, ach, normal from same patient | Tran-scripts | (MMP-2, MMP-9, MMP-13) | MMP-3 | Collagen I, Collagen III, MMP-1 | RT-PCR normalised to 18s rRNA | 96 |
|  |  | Peroperative, ach, normal, n=3, 58 (25-78) |  | Collagen I, Collagen III, (MMP-2, MMP-9, MMP-13) | MMP-3 | MMP-1 | Only results from gene expression study are included in this table |  |
| **Eriksen, 2002**[64] | Peroperative, ach, rupture, n=10, 38 (40-48) | Peroperative, ach, normal from same patient (two control sites) | Proteins | Collagen III | - | Procollagen I, procollagen III |  | 79 |
|  |  | Cadaver, ach, normal, n=6, 43 (13-56) |  | Collagen III | Collagen | Collagen I |  |  |
| **Fenwick, 2001**[65] | Peroperative, ach, tendinopathy, n=7, 38.4 (29-49) | Peroperative, ach, normal from same patient | Proteins | TGF-β2 | - | TGF-β2RII | Quantification of percentage of positively stained cells | 81 |
|  |  | Cadaver, ach, normal, n=4, 75.5 (56-93) |  |  |  |  |  |  |
| **Ireland, 2001**[66] | Peroperative, ach, chronic painful, n=11, 33 (30-35) | Peroperative, ach, normal from same patient | Tran-scripts | Collagen I, Collagen III | MMP-3 | MMP-1, MMP-2, TIMP-3 | RT-PCR normalised to GADPH | 79 |
|  |  |  | Proteins | - | MMP-3 | - | Results from cDNA arrays are not included in this table |  |
| **Jones, 2006**[67] | II: Peroperative, ach, chronic painful, n=9, 45 (33-59) | I: Cadaver, ach, normal n=11, 49 (20-76) | Tran-scripts | ADAM-12, MMP-11,-16,-23, ADAMTS-2, -3 | MMP-3,-10,-12,-27, TIMP-3, ADAMTS-5 | ADAM-17, MMP-2,-13,-15, ADAMTS-1,-6,-8,-9,-12,-15,-16,-18,-19,-20 | RT-PCR normalised to 18s rRNA | 79 |
|  | III: Peroperative, ach, rupture, n=12, 42 (25-53) | I |  | ADAM-8, ADAM-12, MMP-1, MMP-9, MMP-14, MMP-19, MMP-25, TIMP-1, ADAMTS-4 | MMP-3, -7, -24,-28, TIMP-2,-3, -4, ADAMTS-7 |  | ADAMTS-1 and -10, MMP-15 and -25, and TIMP-3 expression tended to decrease with increasing age |  |
|  | III | II |  | ADAM-8,-12MMP-1,-8,-10,-12,-19,-25, ADAMTS-4, TIMP-1 | MMP-7,-16,-23,-24,-28, TIMP-2,-3,-4, ADAMTS-2,-3,-17 |  |  |  |
| **Karousou, 2008**[68] | Peroperative, ach, rupture, n=19, 37.3 (25-58) | Peroperative, ach, normal from same patient | Tran-scripts | Collagen I, MMP-2, TIMP-1, TIMP-2, decorin, versican | - | Collagen IX | RT-PCR normalised to GADPH | 88 |
|  |  |  | Proteins | MMP-2, MMP-9 | - | - | MMP-2 and -9 were also examined by zymography |  |
| **Pajala, 2009**[69] | Peroperative, ach, rupture, n=10, 38 (30-48) | Peroperative, ach, normal from same patient (two control sites) | Proteins | Collagen III, procollagen III | - | TNC, procollagen I |  | 69 |
| **Pingel, 2012**[70] | Peroperative, ach, tendinopathy, n=16, n.r. (n.r.) | Peroperative, ach, normal from same patient | Tran-scripts | Collagen I, Collagen III, fibronectin, TNC, TGF-β1, fibromodulin, MMP-2, MMP-9, TIMP-2 | (Decorin), bFGF, cmet* | Versican, CTGF, VEGF-A1, IGF-1, COX-1, IL-1R, CCL-2, scleraxis, tenomodulin, LOX [HGF-1, IL-6, IL-1β, ki67] | RT-PCR normalised to GADPH. RPLP0 was used as a reference gene. | 88 |
| **Pufe, 2001**[71] | Peroperative, ach, rupture, n=20, 35 (n.r.) | Cadaver, ach, normal, n=n.r (-) | Proteins | VEGF | - | - |  | 50 |

Abbreviations: ach=Achilles tendon, ADAMTS=a disintegrin and metalloproteinase with thrombostin motifs, AGE=advanced glycation end product, bb=biceps brachii tendon, CB_1_R=Cannabinoid receptor type 1, CCL=chemokine ligand, COX=cyclooxygenase, CSF=colony-stimulating factor, CTGF=connective tissue growth factor, FNRB=fibronectin receptor subunit beta, GADPH=glyceraldehyde 3-phosphate dehydrogenase, G-CSF=granulocyte colony-stimulating factor, GM-CSF=granulocyte macrophage CSF, HGF=hepatocyte growth factor, HSP=heat shock protein, IGF=insulin-like growth factor, IL=interleukin, ILR=interleukin receptor, LOX=lysyl oxidase, MAPKp38=mitogen-activated protein kinase p38, MMP=matrix metalloproteinase, n.r.=not reported, TGF-β=transforming growth factor-β, TIMP=tissue inhibitor of metalloproteinases, TNC=tenascin C, VEGF=vascular endothelial growth factor. * synonomous with HGF receptor.
