# Supplementary material for: Proteomics Perspectives in Rotator Cuff Research: A Systematic Review of Gene Expression and Protein Composition in Human Tendinopathy
Source: PLoS One. 2015 Apr 16;10(4):e0119974. doi: 10.1371/journal.pone.0119974 (PMC4400011; doi:10.1371/journal.pone.0119974)
Supplement: S3 Table — None of the studies that quantified proteins used proteomics technologies. Two authors in same row indicate that the same patient and control populations were used in the two studies; () = non-significant trend. (DOCX) [file pone.0119974.s004.docx]

**S3 Table. Gene expression and protein composition in various tendinopathies.** None of the studies that quantified proteins used proteomics technologies. Two authors in same row indicate that the same patient and control populations were used in the two studies; ()=non-significant trend.

|  |  | |  |  | | |  | |
| --- | --- | --- | --- | --- | --- | --- | --- | --- |
|  | **Sample setting, anatomical site of sample, diagnosis, number of patients (n), mean age (range)** | |  | **Direction of change of target tendon components** | | |  | |
|  |  | |  |  | | |  | |
|  |  |  |  |  |  |  |  |  |
| **First author, year** | **Patient samples** | **Control samples** | **Method** | **Up** | **Down** | **No difference** | **Comment** | **Quality** |
|  |  |  |  |  |  |  |  | **Score** |
| **Bridgeman, 2010**[72] | Peroperative, pttd, n=8, 52.4 (18-73) | Peroperative, ptt, normal from same patients | Tran-scripts | - | - | ERα, ERβ | RT-PCR normalised to 18s rRNA | 67 |
|  |  | Peroperative, fdl, normal from same patients |  |  |  |  |  |  |
| **Corps, 2012**[73] | Peroperative, pttd, n= 32, n.r. (19-77) | I: Peroperative, fdl, normal from same patients | Tran-scripts | Collagen I, Collagen III, | MMP-3, ADAMTS-5 | Decorin, versican, | Expression of versican was increased in pttd in male | 75 |
|  |  | II: Peroperative, ptt, normal, n=13, n.r. (29-87) |  | aggrecan, biglycan, MMP-2, MMP-13, MMP-23, ADAM-12L |  | ADAMTS-4 | subjects |  |
|  |  | III: Peroperative, fdl, n=6, 56.5 (54-72) |  | Versican | - | - |  |  |
|  |  | I, III |  | - | ADAMTS-1 | - |  |  |
| **Gonçalves-Neto, 2002**[74] | Peroperative, pttd, n=9, n.r. (49-61) | Peroperative, ptt, normal from same patient (4/9) | Proteins | Collagen III, Collagen V | Collagen I | - |  | 79 |
|  |  | Peroperative, ptt, normal, n=1, 48 |  |  |  |  |  |  |
| **Jelinsky, 2011**[86] | Peroperative, rc tear, | Peroperative, normal tendon | Tran- | Collagen I, | MMP-3, MMP-24 | ADAMTSs | The patient samples were | 71 |
|  | n=15, 58.7 (41-66) | (adjacent tendon), same patient | scripts | Collagen III |  | TIMP-2,-3,-4, | pooled in the analyses. 983 |  |
|  | Peroperative, ercb tear, |  |  | MMP-2,-9,-13,- | IL-17D, IL-6R | versican, | transcripts showed a |  |
|  | n=3, 51.7 (48-55) |  |  | 14,-19 |  | decorin, | significantly differential |  |
|  | Peroperative, |  |  | TIMP-1 |  | biglycan, | expression pattern. Only |  |
|  | flexor/pronator, n=2, |  |  | ADAM-12 |  | fibromodulin, | transcripts that have been |  |
|  | (45, 62) |  |  | TNC, periostin, |  | lumican | previously examined in |  |
|  | Peroperative, pt tear, |  |  | fibronectin, |  |  | relation to tendinopathy are |  |
|  | n=2, (32, 41) |  |  | laminin, |  |  | included in this table. Only |  |
|  | Peroperative, bb tear, |  |  | IL13RA2, |  |  | few differentially regulated |  |
|  | n=1, 45 |  |  | OSMR |  |  | cytokines detected. |  |
|  |  |  |  |  |  |  |  |  |
| **Legerlotz, 2012**[85] | I: Peroperative, painful ach, n= 20, 47.3 (n.r.) | IV: Peroperative, ach, normal, n=9, 45.3 (n.r.) | Tran-scripts | Collagen I, COX-2, IL-6 | IL-6R | CNTF, LIF, OSM, VEGF | RT-PCR normalised to Topo-1 and EIF4A2. 18s rRNA was used as an internal control | 83 |
|  | II: Peroperative, ach rupture, n= 18, 46.3 (n.r.) | I, IV |  | VEGF, COX-2, OSM, LIF, IL-6 | IL-6R | CNTF, Collagen I |  |  |
|  | III: Peroperative, pttd, n= 20, 53.9 (n.r.) | V: Peroperative, ptt, normal, n=10, 47.5 (n.r.) |  | Collagen I, CNTF |  | VEGF, COX-2, OSM, IL-6, IL-6R |  |  |
| **Scott, 2008**[84] | Peroperative, ach tendinopathy, n=13, n.r. (n.r.) | Peroperative, ach, normal, n=7, n.r. (n.r.) | Proteins | vGluT2 | - | - | Semiquantitative evaluation | 81 |
|  | Peroperative, pt tendinopathy, n=1, n.r. (n.r.) | Peroperative, pt, normal, n=8, n.r. (n.r.) |  |  |  |  |  |  |

Abbreviations: 18s rRNA=18S ribosomal RNA, ach=Achilles tendon, ADAM=a disintegrin and metalloproteinase, ADAMTS=a disintegrin and metalloproteinase with thrombostin motifs, bb=biceps brachii tendon, CNTF=ciliary neutrophic factor, COX=cyclooxygenase, EIF42=eukaryot translation initiation factor 4A2, ER=estrogen receptor, ercb=extensor carpi radialis brevis, fdl=flexor digitorum longus, IL=interleukin, IL13RA2=interleukin-13-receptor subunit alpha-2, ILR=interleukin receptor, LIF=leukemia inhibitory factor, MMP=matrix metalloproteinase, n.r.=not reported, OSM=oncostatin M, OSMR=OSM receptor, pt=patellar tendon, ptt=posterior tibial tendon, pttd=posterior tibial tendon dysfunction, rc=rotator cuff tendon, TIMP=tissue inhibitor of metalloproteinases, TNC=tenascin C, VEGF=vascular endothelial growth factor, vGluT=vesicular glutamate transporter
