# Supplementary material for: Proteomics Perspectives in Rotator Cuff Research: A Systematic Review of Gene Expression and Protein Composition in Human Tendinopathy
Source: PLoS One. 2015 Apr 16;10(4):e0119974. doi: 10.1371/journal.pone.0119974 (PMC4400011; doi:10.1371/journal.pone.0119974)
Supplement: S2 Appendix — (DOCX) [file pone.0119974.s006.docx]

**S2 Appendix B.** **Search strings used in Embase and Web of Science.**

*Embase*

*((((*(matrix metalloproteinase* or cytokine* or neuropeptide* or glycoprotein* or proteoglycan* or proteo*).af.) OR (exp matrix metalloproteinase/) OR (*inflammation/) OR (*neuropeptide/) OR (*proteome/) OR (*proteomics/) OR (*rna/) OR (*gene expression/) OR (*protein/) OR (*protein expression/) OR (exp extracellular matrix/) OR (exp glycoprotein/) OR (*proteoglycan/) OR (exp *cytokine/) OR (exp *scleroprotein/)) AND ((exp *tendon injury/) OR (exp *tendinitis/) OR (exp *tendon rupture/) OR (exp *rotator cuff rupture/) OR (exp *achilles tendon rupture/) OR ((tendon* or tendini* or tendino* or rotator cuff tear).af.) OR (((tendon and lacerations).af.) AND (tendon*.af.))) AND (human*.af.)) not review*).af.

*Limits: limit 28 to ((Danish or English or Norwegian or Swedish) and yr="1990 -Current")*

*Web of Science*

(Topic=(matrix metalloproteinase*) OR Topic=(glycoprotein*) OR Topic= (proteoglycan*) OR Topic=(scleroprotein*) OR Topic=(extracellular matrix) OR Topic=(extracellular matrix protein*) OR Topic=(cytokine*) OR Topic=(neuropeptide*) OR Topic=(proteom*) OR Topic=(mrna) OR Topic=(gene expression) OR Topic=(protein expression)) AND (Topic=(tendon rupture*) OR Topic=(tendon injur*) OR Topic=(tendini*) OR Topic=(tendino*) OR Topic=(tendon*) OR Topic=(rotator cuff tear*) OR Topic=(rotator cuff rupture*) OR Topic=(achilles tendon tear*) OR Topic=(achilles tendon rupture*) OR Topic=(tendon laceration*) OR Topic=(tendon tear*)) AND (Topic=(human*) OR Topic=(patient*) OR Topic=(cadav*))

*Refined by: Languages=( ENGLISH ) AND Document Types=( ARTICLE ) AND Languages= ( ENGLISH ) Databases=SCI-EXPANDED Timespan=1990-01-01 - 2012-12-21 Lemmatization=On*
