# Supplementary material for: Proteomics Perspectives in Rotator Cuff Research: A Systematic Review of Gene Expression and Protein Composition in Human Tendinopathy
Source: PLoS One. 2015 Apr 16;10(4):e0119974. doi: 10.1371/journal.pone.0119974 (PMC4400011; doi:10.1371/journal.pone.0119974)
Supplement: S3 Appendix — (DOCX) [file pone.0119974.s007.docx]

**S3 Appendix C. Additional specific search string used for specific proteomics search in Medline.**

((((tendon injur* NOT tendon injuries[MeSH]) OR tendon injuries) OR ((tendinopathy OR ((tendino* OR tendini* OR tendon*) NOT medline[sb]))) OR (tendon AND (lacerations OR rupture)) OR rotator cuff tear OR Achilles tendon tear OR patellar tendon tear)) AND (((((((mass spectrom*)) OR (omic*)) OR (omics[MeSH])) OR (mass spectrometry[MeSH])) OR (proteomics[MeSH])) OR (proteom*))
