# Supplementary material for: Proteomics Perspectives in Rotator Cuff Research: A Systematic Review of Gene Expression and Protein Composition in Human Tendinopathy
Source: PLoS One. 2015 Apr 16;10(4):e0119974. doi: 10.1371/journal.pone.0119974 (PMC4400011; doi:10.1371/journal.pone.0119974)
Supplement: S4 Appendix — (DOCX) [file pone.0119974.s008.docx]

**S4 Appendix D. Manual for quality scoring in Table 1 adapted from Kmet, Lee, and Cook’s Standard Quality Assessment Criteria for evaluating primary research papers from a variety of fields[34].**

**1. Was the question/objective sufficiently described?**

**Yes:** Easily identified in the introductory section (or first paragraph of methods section). Specifies all of the following: purpose, subjects/target population, and parameters under investigation. A study purpose that only becomes apparent after studying other parts of the paper is not considered sufficiently described.

**Partial:** Vaguely/incompletely reported, or some information has to be gathered from other parts of the paper than the introduction/background/objective section.

**No:** Not reported or incomprehensible.

**2. Was the study design and choice of experimental methods evident?**

**Yes:** Design easily identified and appropriate to address the study question/objective.

**Partial:** Design and/or study question not clearly identified, but gross inappropriateness is not evident, or design is easily identified, but only partially addresses the study question.

**No:** Design inappropriate to address the study question/objective, or design cannot be identified.

**3. Was selection and characteristics of patients and controls clearly described?**

**Yes:** Selection strategy designed to obtain an unbiased sample of the relevant target population. Inclusion/exclusion criteria clearly described and defined, and sufficient information characterising the participants provided (including age and sex).

**Partial:** Selection methods are not completely described, but no obvious inappropriateness, or selection strategy is not ideal, but did not likely seriously distort results, and/or information on participants is incomplete.

**No:** No information provided.

**4. Where patients and controls comparable on age and sex?**

**Yes:** Comparable on age and sex or paired samples.

**Partial:** Partial comparability on age and sex, or good comparability with insufficient number of controls.

**No:** Patients and controls not comparable.

**5. Was the control tissue adequate?**

**2.** Healthy control tissue from the same joint as the patient samples, or paired samples from same patient and same joint.

**1.** Cadaveric control samples or samples obtained from different joints than the patient samples – or a combination hereof.

**6. Was the sample size appropriate?**

**Yes:** Seems reasonable with respect to the outcome under study and the study design.

**Partial:** Insufficient data to assess sample size.

**No:** Obviously inadequate.

**7. Were the primary outcome measures evident and well-defined?**

**Yes:** Primary outcomes defined (or reference to complete definitions provided) and measured according to reproducible, objective criteria.

**Partial:** Definitions not reported in detail, but probably acceptable. Instrument or mode of assessment not reported.

**No:** Measures not defined, or are inconsistent throughout the paper. Or, measures employ only ill-defined, subjective assessments.

**8. Were the statistical methods described and justified?**

**Yes:** Analyses are described and appropriate.

**Partial:** Analytic methods are not reported and have to be guessed at, but are probably appropriate. Or minor flaws, some tests appropriate, some not, or multiple testing problems not addressed.

**No:** Analysis not described, or obviously inappropriate analytic methods.

**9. Was some estimate of variance reported for main results?**

**Yes:** Appropriate estimate(s) is/are provided.

**Partial:** Undefined expressions, or no specific data given, but insufficient power acknowledged as a problem. Or variance estimates not provided for all main results/outcomes. Or inappropriate variance estimates.

**No:** No estimates provided.

**10. Were results reported in sufficient detail?**

**Yes:** Results include major outcomes and all mentioned secondary outcomes.

**Partial:** Quantitative results reported only for some outcomes.

**No:** Quantitative results are provided, but for a subset only, or ’n’ changes in unexplained ways across the denominator. Or results for some major or mentioned secondary outcomes are only qualitatively reported when quantitative reporting would have been possible.

**11. Were the results validated by use of other methods?**

**Yes:** Other methods were applied to confirm results (e.g. mRNA data obtained by qPCR are validated on protein level by Western Blot).

**Partial:** Other methods have been provided to validate some, but not all results, or validation experiments have been performed, but results are not presented in the paper. Or methods used for validation are poorly described or inappropriate.

**No:** No validation experiments have been performed.

**12. Were the examiners blinded to disease state or other important characteristics?**

**Yes:** Blinding reported.

**Partial:** Blinding reported, but it is not clear who was blinded.

**No:** Blinding would have been possible, but is not reported.

**N/A:** Not applicable (uncontrolled experimental studies etc.).

**13. Were the conclusions supported by the results?**

**Yes:** All of the conclusions are supported by the data (even if analysis was inappropriate). Conclusions are all based on all results relevant to the study question, negative as well as positive. Part of the conclusions may expand beyond the results, if made in addition to rather than instead of those strictly supported by the data, and if including indicators of their interpretative nature.

**Partial:** Some of the major conclusions are supported by the data, some are not. Or speculative interpretations are not indicated as such.

**No:** None or a very small minority of the major conclusions are supported by the data. Or negative findings clearly due to low power are reported as definitive evidence against alternate hypothesis. Or conclusions are missing.
