# Supplementary material for: Proteomics Perspectives in Rotator Cuff Research: A Systematic Review of Gene Expression and Protein Composition in Human Tendinopathy
Source: PLoS One. 2015 Apr 16;10(4):e0119974. doi: 10.1371/journal.pone.0119974 (PMC4400011; doi:10.1371/journal.pone.0119974)
Supplement: S5 Appendix — Information from some of the excluded papers is used in the discussion. (DOCX) [file pone.0119974.s009.docx]

**S5 Appendix E. Reasons for exclusion after full-text reading (n=48).** Information from some of the excluded papers is used in the discussion.

| **First author, year** | **Title** | **Reasons for exclusion** |
| --- | --- | --- |
| Akeson, 2009[[1](#_ENREF_1)] | Evaluation of intra-articular collagenase, TIMP-1, and TNF-alpha levels before and after anterior cruciate ligament reconstruction. | Article in Turkish. |
| Alfredson, 1999[[2](#_ENREF_2)] | In situ microdialysis in tendon tissue: high levels of glutamate, but not prostaglandin E2 in chronic Achilles tendon pain. | Microdialysis study. Proteins/mRNAs not examined in tissues, but in dialysate. |
| Alfredson, 2001[[3](#_ENREF_3)] | Glutamate NMDAR1 receptors localised to nerves in human Achilles tendons. Implications for treatment? | Immunohistochemistry on patient and control samples (NMDAR1 and AChE), but no comparative quantification. Protein content only examined in dialysate. |
| Alfredson, 2001[[4](#_ENREF_4)] | In vivo microdialysis and immunohistochemical analyses of tendon tissue demonstrated high amounts of free glutamate and glutamate NMDAR1 receptors, but no signs of inflammation, in Jumper's knee. | Immunohistochemistry on patient and control samples (NMDAR1 and AChE), but no comparative quantification. Protein content only examined in dialysate. |
| Backman, 2011[[5](#_ENREF_5)] | Endogenous substance P production in the Achilles tendon increases with loading in an in vivo model of tendinopathy - peptidergic elevation preceding tendinosis-like tissue changes. | In vivo rat model. |
| Berglund, 2006[[6](#_ENREF_6)] | Patterns of mRNA expression for matrix molecules and growth factors in flexor tendon injury: differences in the regulation between tendon and tendon sheath. | In vitro rabbit model. Rabbit flexor tendon biopsies were extracted and analysed by RT-PCR. |
| Bjur, 2005[[7](#_ENREF_7)] | The innervation pattern of the human Achilles tendon: studies of the normal and tendinosis tendon with markers for general and sensory innervation. | Immunohistochemistry on patient and control samples (PGP9.5, SP, and CGRP), but no comparative quantification. Protein content only examined in dialysate. |
| Bjur, 2008[[8](#_ENREF_8)] | Presence of a non-neuronal cholinergic system and occurrence of up- and down-regulation in expression of M2 muscarinic acetylcholine receptors: new aspects of importance regarding Achilles tendon tendinosis (tendinopathy). | No comparative quantification between patient and control samples. |
| Bjur, 2008[[9](#_ENREF_9)] | Immunohistochemical and in situ hybridization observations favor a local catecholamine production in the human Achilles tendon. | No comparative quantification between patient and control samples. |
| Bjur, 2009[[10](#_ENREF_10)] | Presence of the neuropeptide Y1 receptor in tenocytes and blood vessel walls in the human Achilles tendon. | NPY, Y1-, and Y2 receptors were examined by immunohistochemistry, but comparative quantification between patient and control samples was either not performed or only performed semiquantitatively using a four-graded scale, and scores were not reported. |
| Cetti, 2003[[11](#_ENREF_11)] | Spontaneous rupture of the Achilles tendon is preceded by widespread and bilateral tendon damage and ipsilateral inflammation: a clinical and histopathologic study of 60 patients. | Calgranulin and neutrophilic elastase examined by immunohistochemistry, but no comparative quantification between patient and control samples. |
| Chaudhury, 2011[[12](#_ENREF_12)] | Torn human rotator cuff tendons have reduced collagen thermal properties on differential scanning calorimetry. | Structural study. No protein/mRNA quantification. |
| Danielson, 2006[[13](#_ENREF_13)] | Immunohistochemical and histochemical findings favoring the occurrence of autocrine/paracrine as well as nerve-related cholinergic effects in chronic painful patellar tendon tendinosis. | M2-receptor, ChAT, VACht, and AChE examined by immunohistochemistry, but comparative quantification between patient and control samples was either not performed or only performed semiquantitatively for specific structures and criteria/methods for this were not described. |
| Danielson, 2006[[14](#_ENREF_14)] | Distribution of general (PGP 9.5) and sensory (substance P/CGRP) innervations in the human patellar tendon. | Only normal patellar tendons were examined. |
| Danielson, 2007[[15](#_ENREF_15)] | In situ hybridization studies confirming recent findings of the existence of a local nonneuronal catecholamine production in human patellar tendinosis. | TH mRNA examined by ISH, but comparative quantification between patient and control samples was only performed semiquantitatively and criteria/methods for this were not described. |
| Danielson, 2007[[16](#_ENREF_16)] | Studies on the importance of sympathetic innervation, adrenergic receptors, and a possible local catecholamine production in the development of patellar tendinopathy (tendinosis) in man. | Several signal substances examined by immunohistochemistry, but comparative quantification between patient and control samples was either not performed or only performed semiquantitatively for specific structures and criteria/methods for this were not described. |
| Danielson, 2007[[17](#_ENREF_17)] | Extensive expression of markers for acetylcholine synthesis and of M2 receptors in tenocytes in therapy-resistant chronic painful patellar tendon tendinosis - a pilot study. | M2-receptor, ChAT, VACht, and AChE examined by immunohistochemistry and ISH, but comparative quantification between patient and control samples was either not performed or only performed semiquantitatively for specific structures and criteria/methods for this were not described. |
| Danielson, 2008[[18](#_ENREF_18)] | Marked sympathetic component in the perivascular innervation of the dorsal paratendinous tissue of the patellar tendon in arthroscopically treated tendinosis patients. | PGP9.5, SP/CGRP, TH/NPY, and α1- and α2 adrenoreceptors were examined by immunohistochemistry, but comparative quantification between patient and control samples was either not performed or only performed semiquantitatively for specific structures and criteria/methods for this were not described. |
| Forsgren, 2005[[19](#_ENREF_19)] | Vascular NK-1 receptor occurrence in normal and chronic painful Achilles and patellar tendons: studies on chemically unfixed as well as fixed specimens. | NK-1 receptor examined by immunohistochemistry, but comparative quantification between patient and control samples was either not performed or only performed semiquantitatively for specific structures and criteria/methods for this were not described. |
| Franklin, 2012[[20](#_ENREF_20)] | Implication of nerve growth factor and substance P in shoulder pain. | Conference abstract. |
| Gaida, 2012[[21](#_ENREF_21)] | Evidence of the TNF-alpha system in the human Achilles tendon: expression of TNF-alpha and TNF receptor at both protein and mRNA levels in the tenocytes. | TNF-α, TNFR1, TNFR2, ki67*, and caspase-3 were examined by ISH and/or immunohistochemistry, but comparative quantification between patient and control samples was either not performed or only performed semiquantitatively using a five-point scale. |
| Goodmurphy, 2003[[22](#_ENREF_22)] | An immunocytochemical analysis of torn rotator cuff tendon taken at the time of repair. | Vascularity staining and staining for procollagen I, but procollagen I comparisons between patient and control samples could not be performed. |
| Gotoh, 1997[[23](#_ENREF_23)] | Significance of granulation tissue in torn supraspinatus insertions: An immunohistochemical study with antibodies against interleukin-1 beta, cathepsin D, and matrix metalloprotease-1. | No quantitative data. |
| Hamada, 1994[[24](#_ENREF_24)] | Localization of mRNA of procollagen alpha 1 type I in torn supraspinatus tendons. In situ hybridization using digoxigenin labeled oligonucleotide probe. | Procollagen I was examined by ISH, but no comparative quantification between patient and control samples. |
| Josza, 1993[[25](#_ENREF_25)] | Amianthoid fibres in ruptured human tendons. | Histological study. No specific proteins/mRNAs examined. |
| Lehto, 1990[[26](#_ENREF_26)] | Fibronectin in the ruptured human Achilles tendon and its paratenon. An immunoperoxidase study. | Fibronectin was examined by PAP-method, but no comparative quantification between patient and control samples. |
| Mafulli, 2000[[27](#_ENREF_27)] | Light microscopic histology of Achilles tendon ruptures: a comparison with unruptured tendons. | Histological study. No specific proteins/mRNAs examined. |
| Mafulli, 2002[[28](#_ENREF_28)] | Ruptured Achilles tendons show increased lectin stainability. | Histological study. Lectin staining was performed, but was only subjected to semiquantitative evaluation using a five-point scale. |
| Mafulli, 2004[[29](#_ENREF_29)] | Similar histopathological picture in males with Achilles and patellar tendinopathy. | Histological study. No specific proteins/mRNAs examined. |
| Mafulli, 2011[[30](#_ENREF_30)] | Marked pathological changes proximal and distal to the site of rupture in acute Achilles tendon ruptures. | Histological study. No specific proteins/mRNAs examined. |
| Matuszewski, 2012[[31](#_ENREF_31)] | Regional variation in human supraspinatus tendon proteoglycans: decorin, biglycan, and aggrecan. | No patient samples. Regional variations in proteoglycan content were examined in normal cadaver tissue samples. |
| Mosier, 1998[[32](#_ENREF_32)] | Pathology of the posterior tibial tendon in posterior tibial tendon insufficiency. | Histological study. No specific proteins/mRNAs examined. |
| Nakama, 2006[[33](#_ENREF_33)] | Interleukin-6-induced activation of signal transducer and activator of transcription-3 in ruptured rotator cuff tendon. | IL-6, IL-6R, and phosphorylated STAT3 examined by RT-PCR, Western Blot, and immunohistochemistry, but subacromial synovium specimens were used as controls. |
| Nakase, 2002[[34](#_ENREF_34)] | Activation of cartilage-derived morphogenetic protein-1 in torn rotator cuff. | Cartilage-derived morphogenic protein-1 was examined by ISH and immunohistochemistry, but no controls were used. Comparisons were made between four locations within the tear samples. |
| Petersen, 2004[[35](#_ENREF_35)] | Expression of VEGFR-1 and VEGFR-2 in degenerative Achilles tendons. | VEGFR1 and VEGFR2 examined by immunohistochemistry and RT-PCR, but no comparative quantification between patient and control samples. |
| Pufe, 2003[[36](#_ENREF_36)] | Mechanical factors influence the expression of endostatin--an inhibitor of angiogenesis--in tendons. | Endostatin and collagen II was examined by immunohistochemistry and ELISA, but no tendinopathic/ruptured tendon samples were included and comparisons were made between adult and fetal tendons. |
| Pufe, 2005[[37](#_ENREF_37)] | The role of vasculature and angiogenesis for the pathogenesis of degenerative tendons disease. | Review article. |
| Riley, 1994[[38](#_ENREF_38)] | Glycosaminoglycans of human rotator cuff tendons: changes with age and in chronic rotator cuff tendinitis. | No specific proteins/mRNAs were examined. |
| Riley, 1996[[39](#_ENREF_39)] | Tenascin-C and human tendon degeneration. | TNC was only examined in relation to anatomical distribution in tendons. No comparative quantification between patient and control samples. |
| Riley, 1996[[40](#_ENREF_40)] | Prevalence and possible pathological significance of calcium phosphate salt accumulation in tendon matrix degeneration. | No specific proteins/mRNAs were examined. Results from collagen analysis were presented in Riley et al (1994), which was included in the review. |
| Riley, 2009[[41](#_ENREF_41)] | Analysis of diseased posterior tibialis tendon specimens reveals common molecular pathogenesis of tendinopathy at different anatomical sites. | Conference abstract. |
| Rui, 2012[[42](#_ENREF_42)] | Expression of chondro-osteogenic BMPs in clinical samples of patellar tendinopathy. | BMP-2,-4 and -7, Sox-9**, osteopontin, osteocalcin, and TRAP markers were examined by immunohistochemistry, but no quantitative data was provided. |
| Samiric, 2004[[43](#_ENREF_43)] | Characterisation of proteoglycans and their catabolic products in tendon and explant cultures of tendon. | In vitro study using bovine tendon. |
| Satomi, 2008[[44](#_ENREF_44)] | Changes in histoanatomical distribution of types I, III and V collagen promote adaptative remodeling in posterior tibial tendon rupture. | Collagen I, III, and V were examined by immunofluorescence, but no comparative quantification of protein content was performed between patient and control samples. |
| Savitskaya, 2011[[45](#_ENREF_45)] | Effect of angiogenesis-related cytokines on rotator cuff disease: the search for sensitive biomarkers of early tendon degeneration. | Cytokine examinations were conducted on human peripheral blood serum. Rotator cuff samples were only examined histologically and no specific proteins/mRNAs were examined. |
| Schubert, 2005[[46](#_ENREF_46)] | Achilles tendinosis is associated with sprouting of substance P positive nerve fibres. | SP and TH were examined by immunohistochemistry, but comparative quantification between patient and control samples was either not performed or only performed semiquantitatively for specific structures (nerve fibres). |
| Tom, 2009[[47](#_ENREF_47)] | Changes in the composition of the extracellular matrix in patellar tendinopathy. | Same article as the paper by Samiric et al (2009), which was included in the review, only indexed under the author’s first name. |
| Yuan, 2002[[48](#_ENREF_48)] | Apoptosis in rotator cuff tendinopathy. | No specific proteins/mRNAs examined. Immunohistochemistry was applied to identify specific cell types (macrophages and fibroblasts). |

* An antigen named ki67. ** A transcription factor named Sox-9.

1. Akesen B, Demirag B, Budak F. [Evaluation of intra-articular collagenase, TIMP-1, and TNF-alpha levels before and after anterior cruciate ligament reconstruction]. Acta Orthop Traumatol Turc. 2009;43: 214-218.

2. Alfredson H, Thorsen K, Lorentzon R. In situ microdialysis in tendon tissue: high levels of glutamate, but not prostaglandin E2 in chronic Achilles tendon pain. Knee Surg Sports Traumatol Arthrosc. 1999;7: 378-381.

3. Alfredson H, Forsgren S, Thorsen K, Fahlstrom M, Johansson H, Lorentzon R. Glutamate NMDAR1 receptors localised to nerves in human Achilles tendons. Implications for treatment? Knee Surg Sports Traumatol Arthrosc. 2001;9: 123-126.

4. Alfredson H, Forsgren S, Thorsen K, Lorentzon R. In vivo microdialysis and immunohistochemical analyses of tendon tissue demonstrated high amounts of free glutamate and glutamate NMDAR1 receptors, but no signs of inflammation, in Jumper's knee. J Orthop Res. 2001;19: 881-886.

5. Backman LJ, Andersson G, Wennstig G, Forsgren S, Danielson P. Endogenous substance P production in the Achilles tendon increases with loading in an in vivo model of tendinopathy - peptidergic elevation preceding tendinosis-like tissue changes. J Musculoskelet Neuronal Interact. 2011;11: 133-140.

6. Berglund M, Reno C, Hart DA, Wiig M. Patterns of mRNA Expression for Matrix Molecules and Growth Factors in Flexor Tendon Injury: Differences in the Regulation Between Tendon and Tendon Sheath. J Hand Surg Am. 2006;31: 1279-1287.

7. Bjur D, Alfredson H, Forsgren S. The innervation pattern of the human Achilles tendon: Studies of the normal and tendinosis tendon with markers for general and sensory innervation. Cell Tissue Res. 2005;320: 201-206.

8. Bjur D, Danielson P, Alfredson H, Forsgren S. Presence of a non-neuronal cholinergic system and occurrence of up- and down-regulation in expression of M2 muscarinic acetylcholine receptors: new aspects of importance regarding Achilles tendon tendinosis (tendinopathy). Cell Tissue Res. 2008;331: 385-400.

9. Bjur D, Danielson P, Alfredson H, Forsgren S. Immunohistochemical and in situ hybridization observations favor a local catecholamine production in the human Achilles tendon. Histol Histopathol. 2008;23: 197-208.

10. Bjur D, Alfredson H, Forsgren S. Presence of the neuropeptide Y1 receptor in tenocytes and blood vessel walls in the human Achilles tendon. Br J Sports Med. 2009;43: 1136-1142.

11. Cetti R, Junge J, Vyberg M. Spontaneous rupture of the Achilles tendon is preceded by widespread and bilateral tendon damage and ipsilateral inflammation: a clinical and histopathologic study of 60 patients. Acta Orthop Scand. 2003;74: 78-84.

12. Chaudhury S, Holland C, Porter D, Tirlapur UK, Vollrath F, Carr AJ. Torn human rotator cuff tendons have reduced collagen thermal properties on differential scanning calorimetry. J Orthop Res. 2011;29: 1938-1943.

13. Danielson P, Alfredson H, Forsgren S. Immunohistochemical and histochemical findings favoring the occurrence of autocrine/paracrine as well as nerve-related cholinergic effects in chronic painful patellar tendon tendinosis. Microsc Res Tech. 2006;69: 808-819.

14. Danielson P, Alfredson H, Forsgren S. Distribution of general (PGP 9.5) and sensory (substance P/CGRP) innervations in the human patellar tendon. Knee Surg Sports Traumatol Arthrosc. 2006;14: 125-132.

15. Danielson P, Alfredson H, Forsgren S. In situ hybridization studies confirming recent findings of the existence of a local nonneuronal catecholamine production in human patellar tendinosis. Microsc Res Tech. 2007;70: 908-911.

16. Danielson P, Alfredson H, Forsgren S. Studies on the importance of sympathetic innervation, adrenergic receptors, and a possible local catecholamine production in the development of patellar tendinopathy (tendinosis) in man. Microsc Res Tech. 2007;70: 310-324.

17. Danielson P, Andersson G, Alfredson H, Forsgren S. Extensive expression of markers for acetylcholine synthesis and of M2 receptors in tenocytes in therapy-resistant chronic painful patellar tendon tendinosis - a pilot study. Life Sci. 2007;80: 2235-2238.

18. Danielson P, Andersson G, Alfredson H, Forsgren S. Marked sympathetic component in the perivascular innervation of the dorsal paratendinous tissue of the patellar tendon in arthroscopically treated tendinosis patients. Knee Surg Sports Traumatol Arthrosc. 2008;16: 621-626.

19. Forsgren S, Danielson P, Alfredson H. Vascular NK-1 receptor occurrence in normal and chronic painful Achilles and patellar tendons: studies on chemically unfixed as well as fixed specimens. Regul Pept. 2005;126: 173-181.

20. Franklin S, Carr A. Implication of nerve growth factor and substance P in shoulder pain. Osteoarthritis Cartilage. 2012;20: S260.

21. Gaida JE, Bagge J, Purdam C, Cook J, Alfredson H, Forsgren S. Evidence of the TNF-alpha System in the Human Achilles Tendon: Expression of TNF-alpha and TNF Receptor at both Protein and mRNA Levels in the Tenocytes. Cells Tissues Organs. 2012;196: 339-352.

22. Goodmurphy CW, Osborn J, Akesson EJ, Johnson S, Stanescu V, Regan WD. An immunocytochemical analysis of torn rotator cuff tendon taken at the time of repair. J Shoulder Elbow Surg. 2003;12: 368-374.

23. Gotoh M, Hamada K, Yamakawa H, Tomonaga A, Inoue A, Fukuda HO. Significance of granulation tissue in torn supraspinatus insertions: An immunohistochemical study with antibodies against interleukin-1 beta, cathepsin D, and matrix metalloprotease-1. J Orthop Res. 1997;15: 33-39.

24. Hamada K, Okawara Y, Fryer JN, Tomonaga A, Fukuda H. Localization of mRNA of procollagen alpha 1 type I in torn supraspinatus tendons. In situ hybridization using digoxigenin labeled oligonucleotide probe. Clin Orthop Relat Res. 1994: 18-21.

25. Jozsa L, Balint JB, Kannus P. Amianthoid fibres in ruptured human tendons. Pathol Res Pract. 1993;189: 204-208.

26. Lehto M, Jozsa L, Kvist M, Jarvinen M, Balint BJ, Reffy A. Fibronectin in the ruptured human Achilles tendon and its paratenon. An immunoperoxidase study. Ann Chir Gynaecol. 1990;79: 72-77.

27. Maffulli N, Barrass V, Ewen SWB. Light microscopic histology of achilles tendon ruptures: A comparison with unruptured tendons. Am J Sports Med. 2000;28: 857-863.

28. Maffulli N, Waterston SW, Ewen SW. Ruptured Achilles tendons show increased lectin stainability. Med Sci Sports Exerc. 2002;34: 1057-1064.

29. Maffulli N, Testa V, Capasso G, Ewen SW, Sullo A, Benazzo F, et al. Similar histopathological picture in males with Achilles and patellar tendinopathy. Med Sci Sports Exerc. 2004;36: 1470-1475.

30. Maffulli N, Longo UG, Maffulli GD, Rabitti C, Khanna A, Denaro V. Marked pathological changes proximal and distal to the site of rupture in acute Achilles tendon ruptures. Knee Surg Sports Traumatol Arthrosc. 2011;19: 680-687.

31. Matuszewski PE, Chen YL, Szczesny SE, Lake SP, Elliott DM, Soslowsky LJ, et al. Regional Variation in Human Supraspinatus Tendon Proteoglycans: Decorin, Biglycan, and Aggrecan. Connect Tissue Res. 2012;53: 343-348.

32. Mosier SM, Lucas DR, Pomeroy G, Manoli IA. Pathology of the posterior tibial tendon in posterior tibial tendon insufficiency. Foot Ankle Int. 1998;19: 520.

33. Nakama K, Gotoh M, Yamada T, Mitsui Y, Yasukawa H, Imaizumi T, et al. Interleukin-6-induced activation of signal transducer and acitvator of transcription-3 in ruptured rotator cuff tendon. J Int Med Res. 2006;34: 624-631.

34. Nakase T, Sugamoto K, Miyamoto T, Tsumaki N, Luyten FP, Inui H, et al. Activation of cartilage-derived morphogenetic protein-1 in torn rotator cuff. Clin Orthop Relat Res. 2002: 140-145.

35. Petersen W, Pufe T, Zantop T, Tillmann B, Tsokos M, Mentlein R. Expression of VEGFR-1 and VEGFR-2 in degenerative Achilles tendons. Clin Orthop Relat Res. 2004: 286-291.

36. Pufe T, Petersen W, Kurz B, Tsokos M, Tillmann B, Mentlein R. Mechanical factors influence the expression of endostatin--an inhibitor of angiogenesis--in tendons. J Orthop Res. 2003;21: 610-616.

37. Pufe T, Petersen WJ, Mentlein R, Tillmann BN. The role of vasculature and angiogenesis for the pathogenesis of degenerative tendons disease. Scand J Med Sci Sports. 2005;15: 211-222.

38. Riley GP, Harrall RL, Constant CR, Chard MD, Cawston TE, Hazleman BL. Glycosaminoglycans of human rotator cuff tendons: changes with age and in chronic rotator cuff tendinitis. Ann Rheum Dis. 1994;53: 367-376.

39. Riley GP, Harrall RL, Cawston TE, Hazleman BL, Mackie EJ. Tenascin-C and human tendon degeneration. Am J Pathol. 1996;149: 933-943.

40. Riley GP, Harrall RL, Constant CR, Cawston TE, Hazleman BL. Prevalence and possible pathological significance of calcium phosphate salt accumulation in tendon matrix degeneration. Ann Rheum Dis. 1996;55: 109-115.

41. Riley G, Corps A, Avery N, Curry V, Robinson A, Harrall R, et al. Analysis of diseased posterior tibialis tendon specimens reveals common molecular pathogenesis of tendinopathy at different anatomical sites. Rheumatology (Oxford). 2009;48: i9.

42. Rui YF, Lui PP, Rolf CG, Wong YM, Lee YW, Chan KM. Expression of chondro-osteogenic BMPs in clinical samples of patellar tendinopathy. Knee Surg Sports Traumatol Arthrosc. 2012;20: 1409-1417.

43. Samiric T, Ilic MZ, Handley CJ. Characterisation of proteoglycans and their catabolic products in tendon and explant cultures of tendon. Matrix Biol. 2004;23: 127-140.

44. Satomi E, Teodoro WR, Parra ER, Fernandes TD, Velosa APP, Capelozzi VL, et al. Changes in histoanatomical distribution of types I, III and V collagen promote adaptative remodeling in posterior tibial tendon rupture. Clinics. 2008;63: 9-14.

45. Savitskaya YA, Izaguirre A, Sierra L, Perez F, Cruz F, Villalobos E, et al. Effect of angiogenesis-related cytokines on rotator cuff disease: The search for sensitive biomarkers of early tendon degeneration. Clin Med Insights Arthritis Musculoskelet Disord. 2011;4: 43-53.

46. Schubert TE, Weidler C, Lerch K, Hofstadter F, Straub RH. Achilles tendinosis is associated with sprouting of substance P positive nerve fibres. Ann Rheum Dis. 2005;64: 1083-1086.

47. Tom S, Parkinson J, Ilic MZ, Cook J, Feller JA, Handley CJ. Changes in the composition of the extracellular matrix in patellar tendinopathy. Matrix Biol. 2009;28: 230-236.

48. Yuan J, Murrell GA, Wei AQ, Wang MX. Apoptosis in rotator cuff tendonopathy. J Orthop Res. 2002;20: 1372-1379.
