# Supplementary material for: Proteomics Perspectives in Rotator Cuff Research: A Systematic Review of Gene Expression and Protein Composition in Human Tendinopathy
Source: PLoS One. 2015 Apr 16;10(4):e0119974. doi: 10.1371/journal.pone.0119974 (PMC4400011; doi:10.1371/journal.pone.0119974)
Supplement: S6 Appendix — N/A = not applicable. (DOCX) [file pone.0119974.s010.docx]

**S6 Appendix F. Details on quality scoring of included studies.** N/A=not applicable.

Rotator cuff tendinopathy

| **First author, year** | **1** | **2** | **3** | **4** | **5** | **6** | **7** | **8** | **9** | **10** | **11** | **12^a^** | **13** | **Total** |
| --- | --- | --- | --- | --- | --- | --- | --- | --- | --- | --- | --- | --- | --- | --- |
| Bank, 1999[35] | 2 | 2 | 1 | 0 | 1 | 2 | 2 | 1 | 2 | 2 | 0 | N/A | 2 | 71 |
| Benson, 2009[36] | 2 | 2 | 1 | 0 | 1 | 2 | 2 | 2 | 1 | 2 | 1 | 0 | 2 | 69 |
| Chaudhury, 201 [37] | 2 | 2 | 1 | 0 | 2 | 1 | 2 | 2 | 0 | 1 | 1 | 0 | 2 | 62 |
| Hamada, 1997[38] | 2 | 1 | 1 | 0 | 2 | 1 | 2 | 2 | 2 | 2 | 1 | 0 | 1 | 65 |
| Joseph, 2009[39] | 2 | 2 | 1 | 2 | 2 | 2 | 1 | 2 | 2 | 2 | 0 | N/A | 2 | 83 |
| Lakemeier, 2010[41]  Lakemeier, 2011[40] | 2 | 2 | 1 | 1 | 2 | 2 | 2 | 2 | 2 | 2 | 0 | 0 | 2 | 77 |
| Lakemeier, 2010[42]  Lakemeier, 2010[43] | 2 | 2 | 1 | 1 | 2 | 2 | 2 | 2 | 2 | 2 | 0 | 0 | 2 | 77 |
| Lo, 2004[45] | 2 | 2 | 1 | 1 | 1 | 2 | 2 | 2 | 2 | 2 | 1 | N/A | 2 | 83 |
| Lo, 2005[44] | 2 | 2 | 1 | 1 | 1 | 2 | 2 | 2 | 2 | 2 | 0 | N/A | 2 | 79 |
| Lundgreen, 2011[46] | 2 | 2 | 2 | 1 | 1 | 2 | 2 | 2 | 2 | 1 | 0 | 0 | 2 | 73 |
| Millar, 2008[18]  Millar, 2009[47] | 2 | 2 | 1 | 1 | 2 | 2 | 2 | 2 | 2 | 2 | 2 | 0 | 2 | 85 |
| Millar, 2012[48] | 2 | 1 | 2 | 1 | 2 | 2 | 2 | 2 | 2 | 1 | 0 | 2 | 2 | 81 |
| Oliva, 2009[49] | 1 | 1 | 1 | 1 | 1 | 2 | 2 | 0 | 0 | 1 | 2 | 0 | 2 | 54 |
| Qi[85] | 2 | 2 | 0 | N/A | 1 | 2 | 2 | 2 | 2 | 2 | 2 | N/A | 2 | 86 |
| Riley, 1994[50] | 2 | 2 | 1 | 0 | 1 | 2 | 2 | 2 | 2 | 2 | 0 | N/A | 2 | 75 |
| Riley, 2002[51] | 2 | 2 | 1 | 0 | 1 | 2 | 2 | 2 | 2 | 2 | 1 | N/A | 2 | 79 |
| Shindle, 2011[52] | 2 | 2 | 2 | 1 | 2 | 2 | 2 | 2 | 2 | 2 | 1 | N/A | 2 | 92 |
| Shirachi, 2011[53] | 2 | 2 | 2 | 0 | 1 | 1 | 2 | 2 | 1 | 1 | 2 | N/A | 2 | 75 |
| Singaraju, 2008[54] | 1 | 1 | 1 | 0 | 1 | 0 | 1 | 0 | 0 | 1 | 1 | 0 | 1 | 31 |
| Tillander, 2002[55] | 2 | 2 | 1 | 0 | 2 | 2 | 2 | 2 | 0 | 1 | 0 | 2 | 2 | 69 |
| Tomonaga, 2000[84] | 1 | 2 | 1 | 0 | 2 | 1 | 2 | 1 | 0 | 1 | 0 | 0 | 1 | 46 |
| Wang, 2001[83] | 2 | 1 | 0 | 0 | 2 | 1 | 2 | 2 | 1 | 2 | 2 | 0 | 2 | 65 |

Achilles tendinopathy

| **First author, year** | **1** | **2** | **3** | **4** | **5** | **6** | **7** | **8** | **9** | **10** | **11** | **12** | **13** | **Total** |
| --- | --- | --- | --- | --- | --- | --- | --- | --- | --- | --- | --- | --- | --- | --- |
| Alfredson, 2003[71] | 2 | 2 | 1 | 2 | 2 | 1 | 2 | 1 | 0 | 0 | 2 | N/A | 2 | 71 |
| Bjorklund, 2011[72] | 2 | 2 | 1 | 1 | 2 | 2 | 2 | 2 | 2 | 2 | 1 | 2 | 2 | 88 |
| Corps, 2004[73] | 2 | 2 | 1 | 1 | 1 | 2 | 2 | 2 | 1 | 1 | 0 | N/A | 2 | 71 |
| Corps, 2006[74]  Corps, 2008[75] | 2 | 2 | 1 | 1 | 1 | 2 | 2 | 1 | 1 | 2 | 0 | N/A | 2 | 71 |
| De Mos, 2007[33] | 2 | 2 | 2 | 2 | 2 | 2 | 2 | 2 | 2 | 2 | 1 | N/A | 2 | 96 |
| Eriksen, 2002[76] | 2 | 2 | 2 | 1 | 2 | 2 | 2 | 2 | 1 | 1 | 0 | N/A | 2 | 79 |
| Fenwick, 2001[77] | 2 | 2 | 1 | 1 | 2 | 2 | 2 | 2 | 2 | 2 | 1 | 0 | 2 | 81 |
| Ireland, 2001[78] | 2 | 2 | 1 | 2 | 1 | 2 | 2 | 2 | 0 | 1 | 2 | N/A | 2 | 79 |
| Jones, 2006[79] | 2 | 2 | 1 | 1 | 1 | 2 | 2 | 2 | 2 | 2 | 0 | N/A | 2 | 79 |
| Karousou, 2008[80] | 2 | 2 | 1 | 2 | 2 | 2 | 2 | 1 | 2 | 2 | 1 | N/A | 2 | 88 |
| Pajala, 2009[81] | 2 | 2 | 2 | 2 | 2 | 2 | 2 | 2 | 0 | 1 | 0 | 0 | 1 | 69 |
| Pingel, 2012[82] | 2 | 2 | 1 | 2 | 2 | 2 | 2 | 2 | 2 | 2 | 0 | N/A | 2 | 88 |
| Pufe, 2001[83] | 1 | 2 | 0 | 1 | 0 | 2 | 1 | 0 | 1 | 1 | 2 | 0 | 2 | 50 |

Patellar tendinopathy

| **First author, year** | **1** | **2** | **3** | **4** | **5** | **6** | **7** | **8** | **9** | **10** | **11** | **12** | **13** | **Total** |
| --- | --- | --- | --- | --- | --- | --- | --- | --- | --- | --- | --- | --- | --- | --- |
| Fu, 2002a[75]  Fu, 2002b[76]  Fu, 2007[77] | 2 | 2 | 1 | 2 | 2 | 2 | 2 | 2 | 1 | 1 | 1 | 2 | 2 | 85 |
| Parkinson, 2004[78] | 2 | 2 | 0 | 0 | 2 | 2 | 2 | 2 | 2 | 2 | 0 | N/A | 2 | 75 |
| Samiric, 2009[79] | 2 | 2 | 1 | 2 | 2 | 2 | 2 | 2 | 1 | 2 | 2 | N/A | 2 | 92 |
| Schizas, 2010[80]  Schizas, 2012[81] | 2 | 2 | 2 | 1 | 2 | 2 | 2 | 2 | 2 | 2 | 1 | 2 | 2 | 92 |
| Scott, 2008a[82] | 2 | 2 | 1 | 1 | 2 | 2 | 2 | 2 | 1 | 2 | 2 | 2 | 2 | 88 |
| Scott, 2008b[83] | 2 | 2 | 2 | 2 | 2 | 2 | 2 | 2 | 0 | 1 | 1 | 2 | 2 | 85 |

Other tendinopathies

| **First author, year** | **1** | **2** | **3** | **4** | **5** | **6** | **7** | **8** | **9** | **10** | **11** | **12** | **13** | **Total** |
| --- | --- | --- | --- | --- | --- | --- | --- | --- | --- | --- | --- | --- | --- | --- |
| Bridgeman, 2010[72] | 2 | 2 | 1 | 2 | 2 | 0 | 2 | 2 | 0 | 1 | 0 | N/A | 2 | 67 |
| Corps, 2012[73] | 2 | 2 | 2 | 1 | 2 | 2 | 2 | 2 | 0 | 1 | 0 | N/A | 2 | 75 |
| Goncalves-Neto, 2002[74] | 2 | 2 | 1 | 2 | 2 | 2 | 2 | 2 | 2 | 1 | 0 | N/A | 1 | 79 |
| Jelinsky, 2011[86] | 2 | 1 | 1 | 2 | 2 | 2 | 2 | 2 | 0 | 1 | 0 | N/A | 2 | 71 |
| Legerlotz, 2012[85] | 2 | 2 | 1 | 2 | 1 | 2 | 2 | 2 | 2 | 2 | 0 | N/A | 2 | 83 |
| Scott, 2008[84] | 2 | 2 | 1 | 1 | 2 | 2 | 2 | 2 | 2 | 2 | 1 | 0 | 2 | 81 |
